# Microbiomes and metabolomes of dominant coral reef primary producers illustrate a potential role for immunolipids in marine symbioses

**DOI:** 10.1038/s42003-023-05230-1

**Published:** 2023-08-31

**Authors:** Helena Mannochio-Russo, Sean O. I. Swift, Kirsten K. Nakayama, Christopher B. Wall, Emily C. Gentry, Morgan Panitchpakdi, Andrés M. Caraballo-Rodriguez, Allegra T. Aron, Daniel Petras, Kathleen Dorrestein, Tatiana K. Dorrestein, Taylor M. Williams, Eileen M. Nalley, Noam T. Altman-Kurosaki, Mike Martinelli, Jeff Y. Kuwabara, John L. Darcy, Vanderlan S. Bolzani, Linda Wegley Kelly, Camilo Mora, Joanne Y. Yew, Anthony S. Amend, Margaret McFall-Ngai, Nicole A. Hynson, Pieter C. Dorrestein, Craig E. Nelson

**Affiliations:** 1 Skaggs School of Pharmacy and Pharmaceutical Sciences, University of California, San Diego, La Jolla, CA 92093 USA; 2 Department of Biochemistry and Organic Chemistry, Institute of Chemistry, São Paulo State University, Araraquara, SP 14800-060 Brazil; 3 Daniel K. Inouye Center for Microbial Oceanography: Research and Education, Department of Oceanography and Sea Grant College Program, University of Hawaiʻi at Mānoa, Honolulu, HI 96822 USA; 4 Pacific Biosciences Research Center, University of Hawaiʻi at Mānoa, Honolulu, HI 96822 USA; 5 Ecology Behavior and Evolution Section, Department of Biological Sciences, University of California, San Diego, La Jolla, CA 92093 USA; 6 Collaborative Mass Spectrometry Innovation Center, Skaggs School of Pharmacy and Pharmaceutical Sciences, University of California, San Diego, La Jolla, CA 92093 USA; 7 Department of Chemistry and Biochemistry, University of Denver, Denver, CO 80210 USA; 8 Cluster of Excellence “Controlling Microbes to Fight Infections” (CMFI), University of Tuebingen, Tuebingen, Germany; 9 University City High School, San Diego, CA 92122 USA; 10 Marine Option Program, University of Hawaiʻi at Mānoa, Honolulu, HI 96822 USA; 11 Hawaiʻi Sea Grant College Program, University of Hawaiʻi at Mānoa, Honolulu, HI 96822 USA; 12 School of Biological Sciences, Georgia Institute of Technology, 311 Ferst Drive, Atlanta, GA 30332 USA; 13 University of Hawaiʻi at Mānoa, Honolulu, HI 96822 USA; 14 Scripps Institution of Oceanography, University of California San Diego, La Jolla, California, CA USA; 15 Geography, University of Hawaiʻi at Mānoa, Honolulu, HI 96822 USA

**Keywords:** Metabolomics, Microbiome, Symbiosis, Chemical ecology

## Abstract

The dominant benthic primary producers in coral reef ecosystems are complex holobionts with diverse microbiomes and metabolomes. In this study, we characterize the tissue metabolomes and microbiomes of corals, macroalgae, and crustose coralline algae via an intensive, replicated synoptic survey of a single coral reef system (Waimea Bay, Oʻahu, Hawaii) and use these results to define associations between microbial taxa and metabolites specific to different hosts. Our results quantify and constrain the degree of host specificity of tissue metabolomes and microbiomes at both phylum and genus level. Both microbiome and metabolomes were distinct between calcifiers (corals and CCA) and erect macroalgae. Moreover, our multi-omics investigations highlight common lipid-based immune response pathways across host organisms. In addition, we observed strong covariation among several specific microbial taxa and metabolite classes, suggesting new metabolic roles of symbiosis to further explore.

## Abstract (web summary)

Hawaiian coral reef organisms (macroalgae, corals, coralline algae) show host-specific correlation of lipids and specific bacterial groups, and distinct microbiomes are observed between calcifiers and erect macroalgae.

## Introduction

Coral reef benthic communities in shallow tropical habitats are dominated by three main types of primary producers: hermatypic reef corals (Cnidaria in the order Scleractinia), crustose coralline algae (CCA; Rhodophyta in the order Corallinales), and various types of macroalgae. In addition to serving as the sources for the majority of primary productivity on reefs^1,2^, these primary producers together control reef accretion through calcification and dissolution, determining habitat and reef architecture crucial for biodiversity^3–5^. The chemical ecology of benthic assemblages has been widely studied for decades, with notable ongoing advances in areas such as allelopathic interactions between corals and algae^6,7^, composition and bioavailability of dissolved organic matter exudates^8–13^, chemical communications required for symbioses within the coral holobiont^12,14,15^, and signaling compounds produced by CCA and/or their microbial consortia that act as key larval settlement cues for corals and other invertebrates^16^. Together, they act as hosts for a diverse community of microbial taxa on coral reefs, which includes both important primary producers like cyanobacteria and consumers such as heterotrophic bacteria that are capable of recycling dissolved organic matter.

Current methods in untargeted metabolomics^17^ have facilitated the rapid analysis of thousands of known and unknown compounds from hundreds to thousands of samples^18^, which allows comparative metabolomics to investigate how the chemical ecology of organisms vary among species^19^. Identifying compound classes that are either shared or distinct among species is the first step to understanding the evolution and function of these compounds within and across ecosystems. Moreover, constraining and contextualizing the chemical milieu of a “healthy” organism is important for developing a baseline against which to chemically interrogate an organism for signs of stress or disease^20–22^. Comparing the metabolomes of organisms that are critical to ecosystem functioning (i.e., ecosystem engineers) is poised to become a crucial component of Ecosystem- and Resilience-Based Management^23,24^. Comparative metabolomics will help define and characterize the chemical crosstalk that controls the biogeochemistry and function of critical marine habitats like coral reefs.

The greatest diversity of functional genes in macroorganisms is found in their microbiomes: the collection of symbiotic unicellular eukarya, bacteria, archaea, and viruses that inhabit the tissues and surfaces of all plants and animals^25^. Linking microbiome structure to the metabolite composition of distinct interacting organisms can reveal the sources and dynamics of metabolites in ecosystems^26^. Whether a given compound or metabolite is associated with a particular clade of microorganisms or a particular host will help us understand symbioses in complex systems where microbial processes are critical. In the case of holobionts, metabolites are co-produced by the intertwined biochemical processes of both host and microbes. As untargeted metabolomics seeks to further annotate and understand the diverse compounds that comprise biological metabolism, every defined association of uncharacterized compounds within a microbe or host advances our understanding of biochemical ecology.

In many cases, the evolutionary history of host organisms can predict microbiome composition^27^. However, on coral reefs, the evolutionary relatedness of benthic primary producers is complicated by multiple endosymbiosis events and convergent evolution. In evolutionary terms, CCA is closely related to non-crustose red algae (e.g., *Jania* sp.) and more distantly related to green algae (e.g., *Halimeda* sp.). Corals, in contrast, are metazoan mixotrophs harboring dinoflagellates (family: Symbiodiniaceae), which are themselves eukaryotes with photosynthetic organelles ultimately derived from brown algae. As a result, corals are able to both consume particulate organic matter and produce fixed carbon through photosynthesis. Metazoans and dinoflagellates have evolved distinct metabolic pathways relative to algae, though some important and ancient lipid biosynthesis pathways are shared^28^. Variation in how benthic primary producers have evolved to receive carbon, synthesize metabolites, and interact with microbes will all contribute to distinct microbiomes and metabolomes.

In addition to the phylogenetic relatedness of host organisms, it is important to consider the functional traits of benthic primary producers that may structure their microbial communities. Traits related to the physical structure, production of microbial food sources, and host immune response are all potential determinants of microbial community composition. Physical structure, including anatomical microhabitats^29,30^, can influence the settlement and persistence of both macro- and microorganisms. Host exudates provide a microbial food source, selecting microbial taxa that are capable of breaking down these compounds^31^. Host immune response, and the corresponding release of antimicrobial compounds, can be activated through multiple signaling pathways. Analysis of tissue samples using untargeted metabolomics can help coral reef biologists understand how host organisms respond to microbial colonization.

**Fig. 1:**
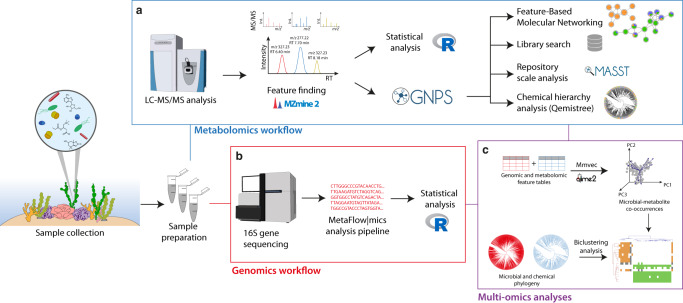
Metabolomic and microbiome workflows followed in this study. The samples were initially collected, prepared, and submitted for the metabolomics and genomics workflow. **a** The metabolomics workflow consisted of data-dependent acquisition (DDA) mode in an LC-ESI-HR-MS/MS instrument in the positive ionization mode, followed by data processing in MZmine2 (feature finding step). The resulting metabolomics feature table was submitted to statistical analysis in R and to the following workflows in the GNPS environment, including Feature-Based Molecular Networking, library searches against annotated spectral databases (available in GNPS) and against datasets in public repositories (MASST), and the Qemistree workflow (in silico annotations and chemical hierarchy analysis). **b** The genomics workflow consisted of 16 S gene sequencing, in which the data were submitted to the Metaflowmics pipeline. The genomic feature table generated was then subjected to statistical analysis in R. **c** Multi-omics analyses (mmvec and biclustering analysis) were employed for data integration. Logos in the figure were obtained from the GNPS, R, Qiime2, and MZmine official websites.

In this study, we sought to synoptically sample specimens of coral, macroalgae, and CCA from a representative tropical reef ecosystem to analyze their metabolomes and microbiomes (workflow in Fig. 1). Previous studies have documented metabolites and microbes across benthic primary producers, but they focused on particular benthic groups (e.g., algae^32–35^ and coral^36–38^), zones of interaction^6,37,39^, or exuded metabolites^31^. The goal of this study was to characterize the microbes and tissue metabolites of all three types of dominant benthic primary producers within a common framework. Tissues were sampled in a broad sense, with each sample representing a homogenate of multiple distinct physiological compartments. Homogenized samples contained surface biofilms, mucus layers, subsurface tissues, and skeletal components (e.g., calcium carbonate substructures), representing a relatively holistic snapshot of the holobiont. We hypothesized that the three types of primary producers would have distinct microbiomes and metabolomes. We predicted that corals would be enriched in known coral bacterial symbionts, such as members of the class Endozoicomonadaceae^40^ and the diazotrophic order Rhizobiales^6,41^, while macroalgae would harbor known copiotrophic microbes, such as Flavobacteriales and Rhodobacterales^42^. We expected that geographic location and evolutionary relatedness of the host would predict microbiome and metabolome composition. Within each producer type, we predicted there would be differentiation among host genera following patterns of phylogenetic relatedness. We focused on collecting discrete host individuals, which led us to exclude from our analysis interwoven species assemblages such as turf algae. Finally, we expected to identify groups of immune signaling compounds across all three primary producer types that could potentially show response to microbial colonization.

**Fig. 2:**
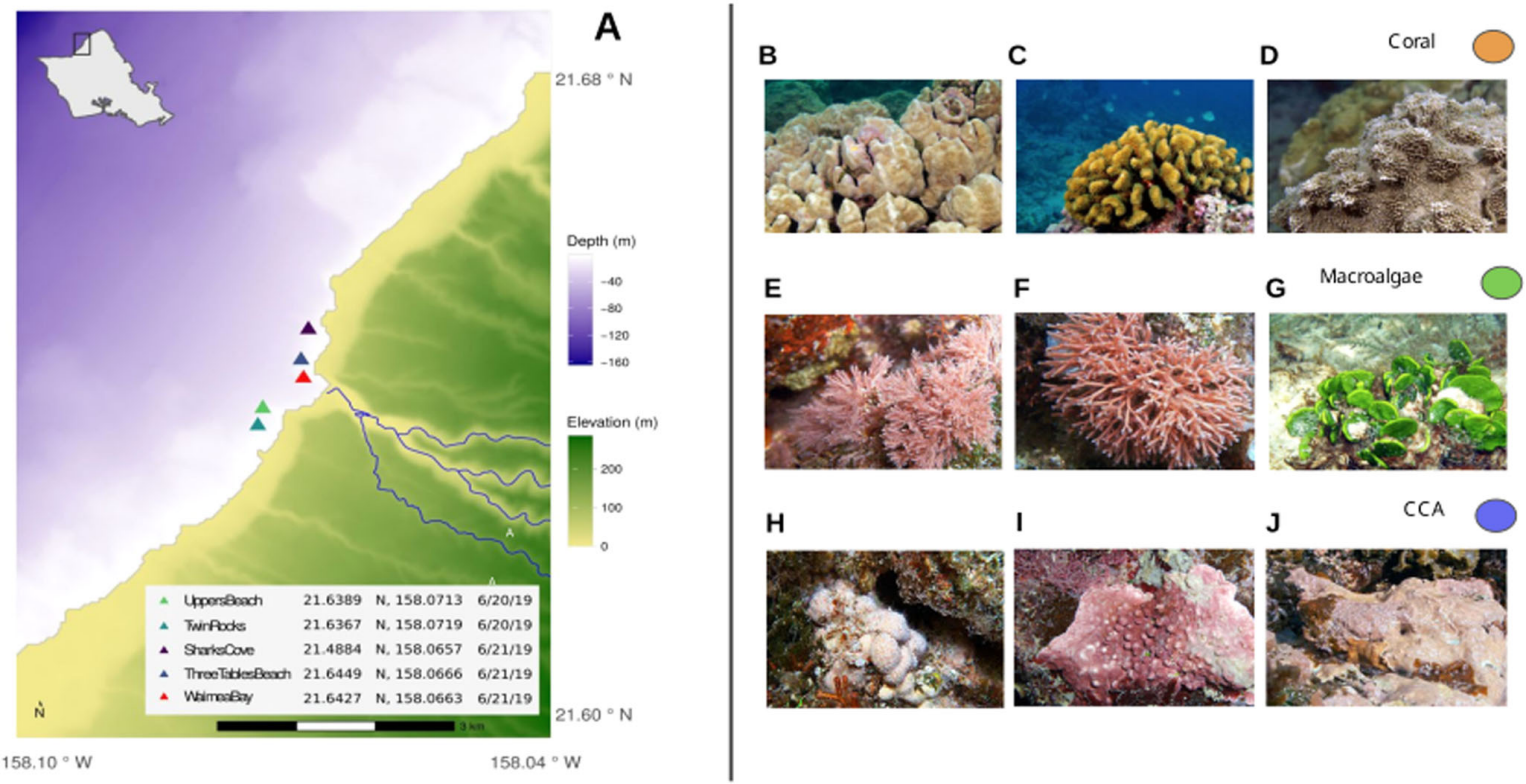
Sampling locations and taxa. **a** Map of sampling sites showing depth and elevation gradients within the study area. Bathymetry data courtesy of Hawaiʻi Mapping Research Group, SOEST, UH Mānoa and elevation data courtesy of the U.S.G.S. National Elevation Dataset. Site locations were collected by handheld GPS. The map was created using the R language for statistical programming (code available in the public GitHub repository associated with this manuscript). **b**–**j** Representative photos of benthic primary producers collected from Waimea Bay, Oʻahu. Scleractinian reef corals (**b**) *Porites lobata*, (**c**) *Pocillopora meandrina*, and (**d**) *Montipora capitata*; erect rhodophytes (**e**) *Jania* and (**f**) *Galaxaura*, and a calcifying chlorophyte (**g**) *Halimeda*; and crustose coralline algae (**h**) *Hydrolithon*, (**i**) *Lithophyllum*, and (**j**) *Hydrolithon* (PC: Keoki and Yuko Stender). For standardization in all of the analyses performed in this work, coral, macroalgae, and CCA samples were defined as orange, green, and blue, respectively. Collection permit: Hawaiʻi State Department of Land and Natural Resources Division of Aquatic Resources Special Activity Permit No. 2020-23. Representative photographs of benthic primary producers were obtained from https://www.marinelifephotography.com with authorization from Keoki Stender.

We further hypothesized that selected microbial taxa would covary with some proportion of metabolites within and among host taxa, allowing us to identify putative microbe-metabolite interactions. Previous metabolomic investigations revealed that lipids play an important role in holobionts and can act as signaling chemicals in inflammatory responses^37^; therefore, we expected to find microbial groups associated with host immune response metabolite pathways. We sought to minimize bias and standardize our analyses by rapidly sampling triplicate biological specimens of multiple species within each category across a wide area of reef over a 2-day period. Our sampling consisted of five benthic sites (Fig. 2) roughly 100 m^2^ designed to span distinct coral reef types flanking a shallow embayment (Waimea Bay, Oʻahu) representative of the high wave-energy north-facing shores of the Hawaiian Archipelago. We analyzed 112 tissue samples from three types of primary producers (41, 45, and 26 samples for CCA, coral, and macroalgae, respectively) to resolve the paired metabolomes (Fig. 1a) and microbiomes (Fig. 1b) of each type, as well as the microbiomes and metabolomes of key genera sampled within each type. We characterized differential abundance of metabolites and microbes, showing clear associations between particular microbial taxa and the primary producer host type. Finally, we uncovered a number of microbe-metabolite covariation patterns (Fig. 1c) that may indicate the source or use of key metabolites within these holobiont systems.

## Results

### Differentiating the metabolomes of dominant coral reef primary producers

Our untargeted metabolomic analysis yielded 11,215 ion features from 112 samples and 15 method blanks. Of these 112 samples, 9 were judged to be poor quality, clustering closely with method blanks in multivariate space (Supplementary Fig. 1), and were removed from subsequent analysis. Of the features in this reduced dataset, 268 were removed as background features (detected only in method blanks), and 2893 were removed as transient features (features detected in less than three samples), leaving a total of 8054 ion features attributable as holobiont tissue-associated metabolites of coral, macroalgae, CCA, and microbial associates. The number of features does not reflect the number of compounds detected in these samples as the same compound can be detected as different adducts, and even in-source fragments can occur. In addition, it should be mentioned that both the extraction protocol and the type of chromatography (liquid chromatography) used in this study highly influence the compounds extracted and detected in these analyses. Therefore, the results that will be described represent only a percentage of the metabolites present in the samples.

**Fig. 3:**
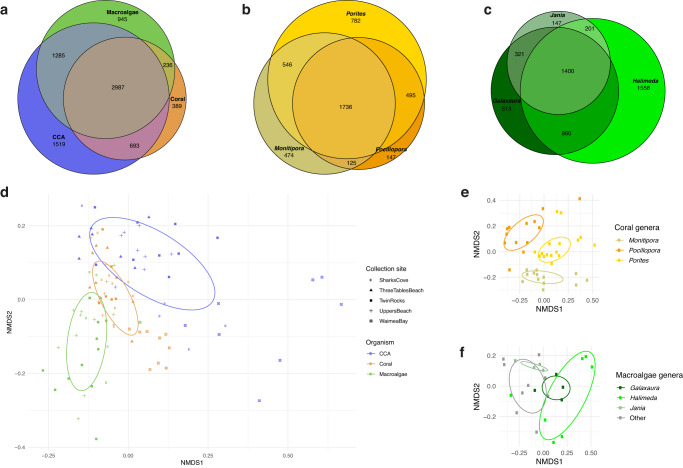
Chemical diversity of coral reef holobiont sample types and genera show the degree of host specificity at both phylum and generic levels. Venn diagrams illustrate chemical richness among hosts: **a** Counts of common and unique ion features among coral (*n* = 42), CCA (*n* = 37), and macroalgae (*n* = 24). **b** Comparison between *Porites* (*n* = 16), *Monitipora* (*n* = 13), and *Pocillopora* (*n* = 13) coral genera and **c** Comparison between *Halimeda* (*n* = 7), *Galaxaura* (*n* = 4), and *Jania* (*n* = 4) macroalgae genera (“other” refers to the less sampled algae genera (*n* = 9)). Ordinations (nonmetric multidimensional scaling of Bray-Curtis dissimilarity matrices from 8054 ion feature relative abundances) illustrate compositional differences among **d** metabolites from each sample type (PERMANOVA *R*^2^ = 0.16; *p* < 0.001) and metabolites from genera of coral (**e**) and macroalgae (**f**).

Of these 8054 features, 35% were unique to only one of the three types of primary producers, while 37% were shared among all three, and 27% were shared among two of the three (Fig. 3a). Corals had the fewest unique ion features (389) while CCA had the most unique ion features (1519). The most shared features were between CCA and macroalgae (1285). We also analyzed the degree of overlap among well-replicated genera within each type of primary producer, showing that coral genera shared a high proportion of metabolites (67%, Fig. 3b) while macroalgae had fewer commonalities among genera (54%, Fig. 3c). Additionally, 31% of the macroalgae features were exclusively detected in *Halimeda* spp., which was the sole green algae (Chlorophyta) representative in the dataset.

We used multivariate approaches to further explore the degree of metabolomic differentiation among benthic primary producers and among genera within each type of primary producer (Fig. 3d–f, Supplementary Fig. 2, Supplementary Data 1). An ordination of tissue samples in multivariate space (Bray-Curtis distance metric) clearly indicated that each primary producer type presented a distinct metabolite profile (Fig. 3d, PERMANOVA *R*^2^ = 0.16; *p* < 0.001). There was roughly equal differentiation between CCA and corals (pairwise PERMANOVA *R*^2^ = 0.112; *p* = 0.001), between CCA and macroalgae (*R*^2^ = 0.122; *p* = 0.001) and between macroalgae and corals (*R*^2^ = 0.130; *p* = 0.001). Differences among the three types of primary producers were greater than the site-to-site variation within each type (PERMANOVA Site *R*^2^ = 0.10; *p* < 0.001) and the interaction between site and type (*R*^2^ = 0.09; *p* < 0.001). We evaluated the degree of dispersion within each primary producer type, which indicated that CCA had the most variable metabolomes across all sites (average distance to median 0.5). CCA dispersion was significantly greater (ANOVA *p* < 0.05) than the dispersion of both coral and macroalgae (average distance to median 0.42 and 0.45), which did not differ significantly from one another (ANOVA *p* = 0.35). The variability in CCA metabolomes was driven in part by higher site-to-site heterogeneity (Fig. 3d). Evaluating the effect of site within each type of primary of primary producer, (Supplementary Fig. 3, Supplementary Data 1), CCA differed the most between sites (*R*^2^ = 0.20, *p* = 0.001), while corals differed the least (*R*^2^ = 0.16, *p* = 0.001), and macroalgae were intermediate (*R*^2^ = 0.18, *p* = 0.002). Within corals, there was clear separation among the genera *Porites*, *Montipora*, and *Pocillopora* (PERMANOVA *R*^2^ = 0.16, *p* = 0.001; Fig. 3e) while macroalgae exhibited variable patterns of heterogeneity among the three genera analyzed (*Halimeda, Galaxaura*, and *Jania*; *R*^2^ = 0.25, *p* = 0.001 Fig. 3f).

We identified specific ion features differentially enriched in corals, macroalgae, and CCA using two methods: random forest (RF) and linear models with correction for multiple comparison (LM) (Supplementary Data 2). Out of 8054 ion features, 128 exhibited strong predictive power (Supplementary Data 2) for discriminating between primary producer types based on RF (Mean Decrease Accuracy scores >2 standard deviations above the mean). In addition, 1160 features differed significantly in relative abundance among the three primary producer types based on LM (FDR-adjusted *p* < 0.001 and mean centered log-ratio in a sample type > 1). Based on our criteria, RF selected a more conservative subset of features associated with the different primary producer types compared to LM. However, almost all of the RF selected features (124 out of 128) were also significantly differentially abundant in sample types as tested by LM. Variable importance in RF was used to identify a smaller set of ion features that were predictive of primary producer type, while the LM analysis highlighted a broader set of differentially abundant features across primary producer types.

### Molecular networking and spectral annotation

**Fig. 4:**
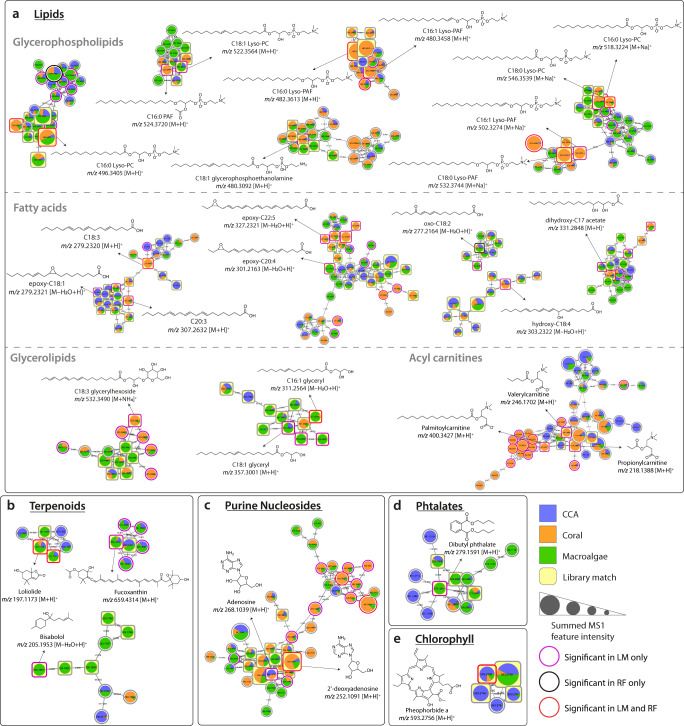
Molecular families comprising features associated with coral reef benthic primary producers. Molecular families are networks of structurally similar ion features: nodes represent tandem mass spectrometry (MS/MS) spectra of distinct ion features and edges represent the MS/MS fragmentation spectral similarity (cosine >0.7). Nodes are labeled by the precursor mass of each feature. Pie charts in each node indicate the relative abundance of metabolites in each benthic primary producer (CCA, coral, and macroalgae). Node sizes are relative to the summed peak areas of the precursor ion in MS1 scans. Information regarding the significance of each feature in Random Forest (RF) and Linear Model (LM) algorithms are shown. Structures represent level 2 annotations (according to the 2007 metabolomics standards initiative^47^) of the primary producer associated features based on the library matches and molecular formula confirmation. **a**–**e** Molecular families are classified as (**a**) lipids, (**b**) terpenoids, (**c**) purine nucleosides, (**d**) phthalates, and (**e**) chlorophyll. The structures show the spectral library matches, and the compounds present in the samples could be their isomers.

To better understand the chemistry of the metabolites detected, we used three informatic approaches to identify and categorize molecular ion features: Feature-Based Molecular Networking^43^ combined with library searches, Qemistree^44^, and MASST^45^, all of which were implemented in the Global Natural Products Social Molecular Networking (GNPS) platform^46^. First, molecular networks were constructed to organize ion features into molecular families and the MS/MS spectra were searched against the GNPS public spectral reference libraries (speclibs). Each spectral match and mass error was evaluated to confirm level 2 annotations according to the Metabolomics Standard Initiative (MSI)^47^. Molecular families containing features that exhibited both high organism association in the statistical analyses (Supplementary Data 2) and matched spectra in the (GNPS) libraries are shown in Fig. 4, while the complete molecular network obtained is shown in Supplementary Fig. 4. The structures shown in Fig. 4 represent the spectral matches obtained from the GNPS libraries. It should be noted that as untargeted mass spectrometry methods are not able to differentiate regio- and stereo-isomers, the position of substitutions and double bounds was not determined.

The molecular families shown in Fig. 4 contained predominantly library matches to lipids, terpenoids, purine nucleosides, and chlorophyll derivatives. Metabolites and molecular families that were statistically associated with primary producer type and differentially enriched in specific primary producer types are described in detail in Supplementary Note 1. All the metabolites with a significant primary producer type association shown in Fig. 4 were differentially abundant in coral and macroalgae samples. Some networks, as in the glycerophospholipids, had multiple features that differentiated between sample types based on RF importance. In addition, matches to phthalates were also retrieved, which could be natural products produced by bacteria^48,49^, or accumulated from plastic contaminants^50^—though contamination from sample collection cannot be ruled out.

Metabolites detected in CCA samples proved to be particularly cryptic. No library matches were observed within the molecular families composed predominantly of features that were abundant in CCA. Several of these metabolites were significantly associated with primary producer type (based on RF and LM) and were enriched in CCA, indicating that CCA may be a source of novel chemical diversity (Supplementary Fig. 5).

### Metabolite in silico annotations and repository-scale analysis

**Fig. 5:**
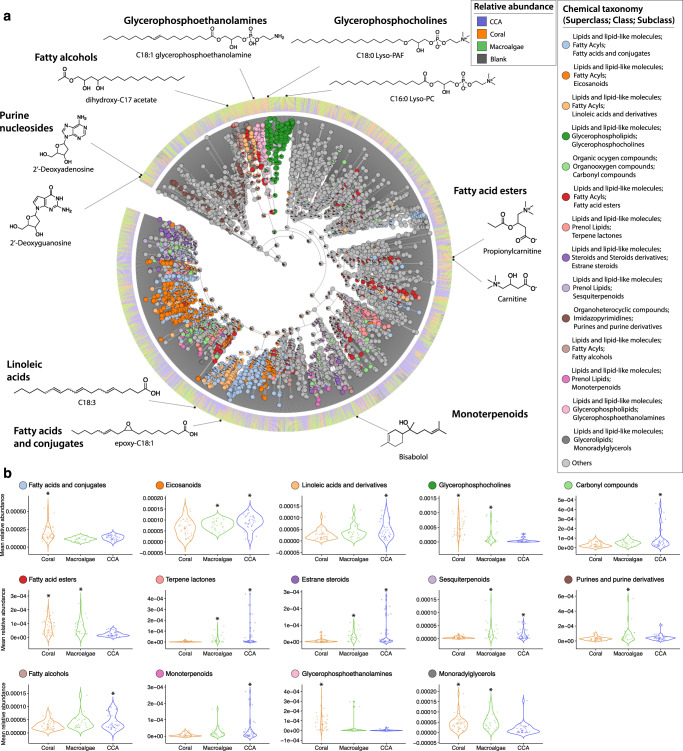
Chemical classification of diverse ion features and subclasses that are differentially associated with each coral reef primary producer. **a** A chemical hierarchy of the predicted molecular fingerprints from the CCA, coral and macroalgae samples. The tree is pruned to keep only fingerprints classified to a subclass level in the ChemOnt ontology using ClassyFire^123^ (total of 3,205 branches). Tree tips of the dendrogram show the annotation of each chemical feature. The internal nodes with pie charts depict the distribution of subclasses with differential abundance among primary producers (ANOVA, *p* < 0.04), and the bar plots at the outer ring indicate the relative abundance of a molecular fingerprint in each primary producer type. The structures shown were retrieved from spectral library matches of the feature-based molecular networking workflow (level 2 or 3 annotations according to the 2007 metabolomics standards initiative^47^), and the compounds present in the samples could be their isomers. b Violin plots display the distribution of each subclass in coral (*n* = 42), macroalgal (*n* = 24), and CCA (*n* = 37) tissue samples.

Out of the 8054 ion features, 5271 fingerprints were obtained by SIRIUS4, ZODIAC, and CSI:FingerID. Only 377 features were direct matches to compounds in spectral libraries (4.6% of total). The dendrogram depicted in Fig. 5a, created using Qemistree, shows the hierarchical classification of a subset of ion features that were classified at the subclass level. Subclasses that exhibited a high correlation with specific benthic primary producers (ANOVA *p* < 0.04) are highlighted and their relative abundance in each type is shown in the violin plots (Fig. 5b). Dendrograms highlighting the most abundant classifications obtained at superclass, class, and subclass levels are shown in Supplementary Fig. 6. Ion features that were predictive of primary producer type, as identified by RF, were mostly unidentified at the subclass level (87 unidentified out of 128 total). The subclasses containing the highest number predictive features were glycerophosphocholines (*n* = 7), fatty acids and conjugates (*n* = 5), and fatty acid esters (*n* = 4).

More than a dozen metabolite subclasses were significantly differentially abundant among the primary producer types (FDR adjusted *p* < 0.05). Some compound subclasses were likely to contain compounds that directly affected microbes. For example, prenol lipids (e.g., terpene lactones, sesquiterpenoids, and monoterpenoids) can have antimicrobial effects and were present at a higher relative abundance in macroalgae and CCA. Some subclasses were likely to contain signaling compounds that could potentially be affected by symbiotic microbes, such as purine derivatives, which had higher relative abundance in macroalgae, and both estrane steroids and carbonyl compounds, which were present at a higher relative abundance in both macroalgae and CCA. Finally, several metabolite subclasses were likely to contain immunolipids relevant to microbial symbiosis. These included fatty acids and conjugates, linoleic acids and derivatives, glycerophosphocholines, and eicosanoids^37,51,52^. Ion features classified as glycerophospholipids (glycerophosphocholines and glycerophosphoethanolamines) and glycerolipids were associated with both coral and macroalgae. The different subclasses of fatty acyls presented variable association with primary producer type. Linoleic acids derivatives and fatty alcohols were observed at a higher relative abundance in CCA, while fatty acids were highly associated with corals. Metabolites classified as eicosanoids were similarly enriched in both macroalgae and CCA, while those classified as fatty acid esters were associated with both macroalgae and coral.

Several molecular families associated with CCA did not result in library matches (Supplementary Fig. 5) or in silico annotations. However, a search of their MS/MS spectra in a repository containing thousands of datasets provided important information about their distribution across various sample types. The fragmentation spectra for all 158 features in these networks were searched against public datasets^45^ (Supplementary Data 3). Only three features from the largest CCA molecular family matched with MS/MS spectra detected in public datasets, both of which were associated with coral reef benthic habitats (MSV000080572—Coral Reef ARMS: 11% of the samples; and MSV000079146—Marine, coral, ARMS: 5% of the samples). In contrast, features of another CCA network matched numerous observed spectra across several public datasets, including environmental samples from coral, including *Porites* sp., as well as fish gut microbiomes, and stromatolites. In addition to environmental samples, these spectra were observed in cultivated bacteria, including cyanobacteria, *Bacillus* sp., and *Streptomyces* sp. Features belonging to three molecular families associated with CCA did not match any spectra published in publicly available datasets.

### Differentiating the microbiomes of dominant coral reef primary producers

**Fig. 6:**
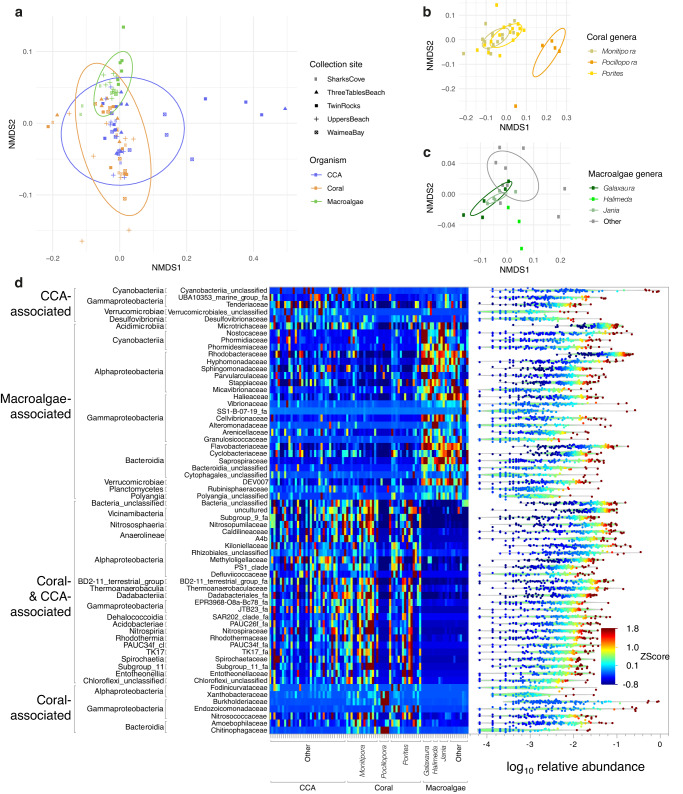
Diversity and holobiont associations of microbial taxa demonstrate clear overlap between coral and CCA microbiomes and distinct macroalgal holobiont communities. **a** Non-metric Multidimensional Scaling (NMDS) of coral (*n* = 35), CCA (*n* = 36), and macroalgae (*n* = 22) samples based on an unweighted UniFrac distance matrix produced from 36,009 Amplicon Sequence Variants (ASVs) (PERMANOVA Sample Type *R*^2^ = 0.16; *p* < 0.001). **b** NMDS plot of coral genera *Porites* (*n* = 15), *Monitipora* (*n* = 15) and *Pocillopora* (*n* = 5). **c** NMDS plot of macroalgae genera *Halimeda* (*n* = 3), *Galaxaura* (*n* = 5), and *Jania* (*n* = 5) (“other” refers to the less sampled algae genera (*n* = 9)). **d** Heatmap shows standardized (z-scored) summed relative abundance for each Family of Bacteria (rows, organized by sample type association and Class) across samples (columns, organized by sample type). Included are all abundant, widespread Bacteria families (found in at least 1/3 of samples with maximum relative abundance >2%) exhibiting significant differences in mean relative abundance among coral, CCA and macroalgae tissue samples. At right is the distribution of relative abundance among the 93 samples for each Family.

Our amplicon sequencing pipeline yielded 36,009 amplicon sequence variants (ASVs) from the 112 samples for which host tissue-associated Bacteria and Archaea were successfully amplified. After filtering out rare ASVs and rarefying samples to an even sequencing depth of 15,000 reads per sample, 4989 ASVs and 93 samples remained in the data set. We used multivariate approaches to explore the degree of microbiome differentiation among CCA, coral, and macroalgae, and the genera associated with those primary producer types (Fig. 6). Tissue samples included surface biofilms, living cells, and calcified structural components. An ordination of tissue samples in multivariate microbiome space (Fig. 6a) indicated that, similar to the metabolome, each primary producer type exhibited a distinct microbial community (PERMANOVA *R*^2^ = 0.16; *p* = 0.001). In contrast to the metabolomes, we found no significant differences in microbiomes composition within sample groups by the site they were collected from (*p* = 0.197) or evidence of site-to-site variation within corals or CCA (*p* = 0.67 and *p* = 0.22, respectively)—Supplementary Fig. 7, Supplementary Data 1. Within the macroalgae, we did see significant site-to-site microbiome variation (*R*^2^ = 0.38; *p* = 0.001). In contrast to the metabolomes, which exhibited roughly equal differentiation among the three primary producer types, the microbiomes of macroalgae differed more from corals (*R*^2^ = 0.19) and from CCA (*R*^2^ = 0.17) than corals and CCA differed from each other (*R*^2^ = 0.06; all pairwise PERMANOVA *p* = 0.001).

As with the metabolomes, multivariate dispersion of microbiomes was highest in CCA (average distance to median 0.182). CCA dispersion was significantly higher than macroalgae (*R*^2^ = 0.112; *p* = 0.024) with corals showing an intermediate level of dispersion (0.168). Overall, dispersion among microbiomes was less than half that of the metabolomes, suggesting significantly greater homogeneity of microbial consortia relative to chemical composition. Similarly to the metabolomes, there was an evident separation of microbiomes among the three most sampled coral genera (PERMANOVA *R*^2^ = 0.34; *p* = 0.001) and macroalgae genera (PERMANOVA *R*^2^ = 0.38; *p* = 0.001) (Fig. 6b, c).

The enrichment patterns of abundant, widespread bacterial families that were statistically associated with particular benthic holobiont primary producers are summarized in Fig. 6d. Many microbial families were differentially abundant in macroalgae (*n* = 25), and showed consistent patterns in relative abundance across the genera sampled. Macroalgae microbiomes were compositionally similar to one another. This is evidenced by the comparatively low dispersion of macroalgal samples observed in the NMDS ordination compared to the dispersion coral and CCA samples (Fig. 6a). Macroalgae were frequently associated with marine copiotrophs, including Sapropsiraceae, Rhodobacteraceae, Flavobacteriaceae, Vibrionaceae, and unclassified Cytophagales. Relatively few microbial families were associated exclusively with coral or CCA (*n* = 7 and *n* = 5). Coral associates included the families Endozoicomonadaceae, common members of the coral ‘core microbiome’^40^, and Nitrosococccaceae, which contains ammonia oxidizing taxa. The families Chitinophagaceae and Burkholderiaceae were also present at a higher relative abundance in coral and have been implicated in coral disease and stress^53,54^, while associates of CCA tended to belong to poorly described groups, including the family Tenderiaceae and Marine Group UBA10353. Cyanobacteria, which are microbial primary producers, were common in microbial communities across the three primary producer types. Eight families of Cyanobacteria were present in more than half of all samples. The majority of these common cyanobacteria were more abundant in macroalgae (Supplementary Fig. 8).

Many families had a dual association with coral and CCA (*n* = 26), underscoring the broad similarity between coral and CCA microbiomes when compared to the microbiome of macroalgae. There was little overlap between the microbial families associated with coral and/or CCA and the families associated with macroalgae. The summed relative abundance of microbial families varied across coral genera. *Pocillopora* harbored a distinct microbial community compared to *Montipora* and *Porites*. Highly abundant microbial associates of both CCA and coral included Kiloniellaceae, Chloroflexi, BD 2-11 Terrestrial group (Gemmatimonadetes), unclassified Rhizobiales, Nitrospiraceae, and Nitrospumilaceae.

### Microbe-metabolite associations

**Fig. 7:**
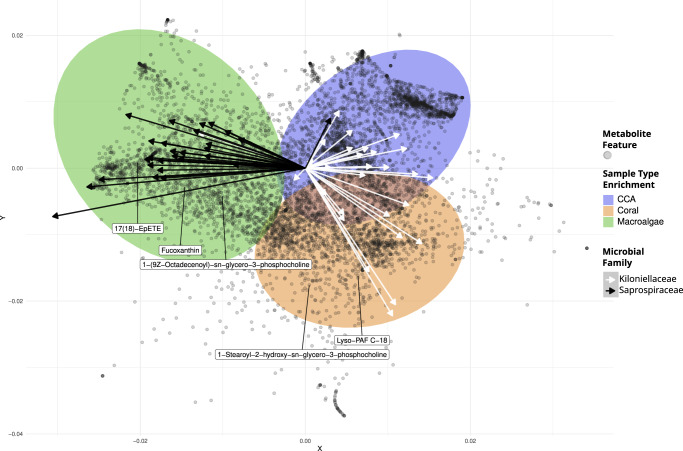
Microbe-metabolite co-occurrence analysis (mmvec) demonstrates holobiont-specific associations between bacterial taxa and ion features. In the biplot, spheres represent metabolites. The axes are the first two principal coordinates learned from the conditional probabilities of microbe-metabolite co-occurrence. Filled ellipses represent the 95% confidence intervals for metabolites that were differentially abundant in a primary producer type based on LM. Arrows represent microbial ASVs belonging to two families, Kiloniellaceae (white, *n* = 28) and Saprospiraceae (black, *n* = 30), which were strongly associated with coral and macroalgae, respectively. Arrows pointing in the direction of metabolites indicate microbe-metabolite co-occurrence. Small angles between arrows indicate microbes co-occurring with each other. The names of the ion features were retrieved from spectral library matches of the feature-based molecular networking workflow, and the compounds present in the samples could be their isomers.

We integrated microbiome and metabolome datasets using the tool mmvec^55^ which implements a neural networking algorithm to predict co-occurrence patterns between microbiome ASVs and ion features. A multivariate ordination of metabolite mmvec scores (Fig. 7) provided a visual tool for understanding the processes that structured microbe-metabolite co-occurrence in the different benthic primary producers. Microbe-metabolite associations proved to be distinct across primary producer types. Metabolites that were associated with each type occupied separate regions of the ordination (colored 95% ellipses). This indicates that the association between a given metabolite and a primary producer type could generally be inferred by looking at the suite of microbes that the metabolite co-occurred with. The PC1 axis separated metabolites enriched in macroalgae from those enriched in coral and CCA, while the PC2 axis separated metabolites enriched in coral from those enriched in CCA and macroalgae. Microbes showed consistent trends in metabolite association at the family level; ASVs in the same family tended to co-occur with similar groups of metabolites. Two exemplary families are shown, illustrating the co-occurrence of algal associated, copiotrophic bacteria (family Saprospiraceae) with algal metabolites, and the co-occurrence of coral associated bacteria (family Kiloniellaceae) with both coral and CCA metabolites (Fig. 7, arrows). Additional family level associations can be found in the Supplementary Fig. 9.

**Fig. 8:**
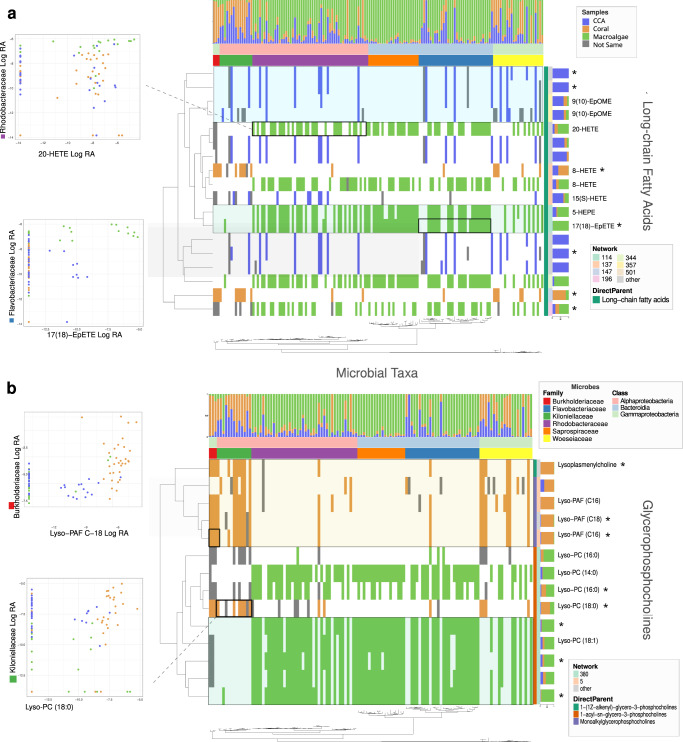
Associations between microbial taxonomic groups and metabolites from two chemical subclasses are associated with specific dominant coral reef benthic producers. Binary biclusters indicate the presence/absence of microbe-metabolite co-occurrence (mmvec) for (**a**) long chain fatty acids and (**b**) glycerophosphocholines (see Fig. 1c). A filled tile indicates that the microbe and metabolite co-occurred. The color of the tile indicates the primary producer type in which the microbe and metabolite co-occurred. Metabolites (rows) are arranged by structural relatedness (Qemistree). Microbes (columns) are arranged by phylogenetic relatedness (16 S FastTree). Metabolites that matched known spectra are annotated with their putative molecular names. Metabolites that were significantly associated with primary producer type are indicated with “*”. Several associations between specific metabolites and microbial families are highlighted. Dot plots show the log relative abundance of microbial families against the log relative abundance of ion features across all samples. The names of the ion features were retrieved from spectral library matches of the feature-based molecular networking workflow, and the compounds present in the samples could be their isomers.

To better visualize co-occurrence patterns across microbe/metabolite pairs, a bicluster graphic was constructed using the R package ComplexHeatmap^56^ (Fig. 8 and Supplementary Fig. 10). This visual analysis links covariation of specific microbes and metabolites with co-enrichment in the tissues of benthic primary producer types. Colored cells in the bicluster indicate positively co-occurring microbe/metabolite pairs that were most abundant in the same primary producer type. Microbial taxa (columns) were arranged by 16 S phylogenetic relatedness, highlighting trends in co-occurrence and sample type abundance across taxonomic groups. Metabolites (rows) were arranged by similarity in chemical structure (annotated in silico using the Qemistree workflow), showing variation in both primary producer enrichment and microbial co-occurrence across closely related chemical compounds. The resulting bicluster (with 1266 microbes and 438 metabolites features retained, Supplementary Fig. 10) highlighted patterns in microbe/metabolite associations that occurred within the different primary producer types. As expected, microbe/metabolite pairs that were most abundant in the same type tended to have high mmvec scores, indicating a high probability of co-occurrence. It should be noted that low and negative mmvec values indicate no relationship, not necessarily a negative correlation.

The biclusters in Fig. 8 show microbe/metabolite associations in two metabolite subclasses: glycerophosphocholines and long-chain fatty acids. These chemical subclasses were differentially abundant in the primary producer types (Figs. 4, 5) and contained features that generated library matches to known immune signaling compounds. To show how microbe/metabolite associations shifted across primary producer types, we selected a set of macroalgae associated families (Rhodobacteraceae, Flavobacteriaceae, and Saprospiraceae) and a set of CCA/coral associated microbial families (Burkholderiaceae, Killoniellaceae). A microbial family that was not associated with any specific type (Woeseiaceae) was included for comparison. The intersecting dendrograms of microbial phylogeny and metabolite structure put co-occurrence patterns into a framework of microbial and chemical relatedness (Fig. 1c). This analysis showed structurally similar lipid based signaling compounds in CCA, coral, and macroalgae associating with different microbial families characteristic of those primary producer types (Fig. 8). The underlying associations between the relative abundance of microbial families and specific ion features are shown as insets in the figure. The inset features include spectral library matches to 20-hydroxyeicosatetraenoic acid (20-HETE), an established immune signaling compound in mammals; 17,18-Epoxy-5,8,11,14-eicosatetraenoic acid (17(18)-epETE), a lipid, abundant in marine organisms, that is a microbially dependent mediator of mammalian allergy, immune, and inflammatory response^57,58^; Lyso-PAF, an immune response compound that, together with PAF, is involved in lipid stress response pathways in both mammals and corals^36,37,59^; and 1-Stearoyl-2-hydroxy-sn-glycero-phosphocoline (Lyso-PC 18:0), a derivative of lysophosphatidylcholine that has been linked to lower cancer rates in humans and has been reported in soft corals^60,61^. In each case, there is a positive co-occurrence relationship between the microbial family and the immune signaling compound.

## Discussion

Coral reef benthic primary producers represent chemically and microbially distinct holobionts. The composition of these organisms in a reef ecosystem affects the chemical compounds and microbial communities that occur on that reef. As benthic communities change, there are corresponding shifts in the biochemical processes occurring on the reef, which are a product of both the host genomes and bacterial metagenomes^62^. Understanding these microbially mediated chemical processes may help to explain the persistence of coral dominated reefs and global trends in coral reef degradation and shifts towards algal dominated systems. A necessary first step is to compare microbes and metabolites that are associated with dominant reef primary producers, including corals, CCA, and macroalgae.

Our multi-omics analyses identified patterns in microbe-metabolite co-occurrence, which pointed towards potential microbe-host interactions in benthic holobionts. Our results largely corroborate our hypothesis that the three dominant benthic primary producers in coral reefs (corals, CCA, and macroalgae) harbor statistically distinct microbiomes and metabolomes. High-throughput chemical and microbial annotations allowed us to compare known immune signaling compounds across benthic holobionts. Correlations between microbial families and organism-associated immune signaling compounds may provide key insights into how host-microbe associations are either maintained or perturbed in reef ecosystems. This approach could be used to characterize multifaceted shifts in coral reef health, for example monitoring levels of copiotrophic microbes associated with macroalgae along with changes in the immune response pathways of both algae and coral. While targeted experiments are necessary to elucidate specific mechanisms of host-microbe interaction, untargeted metabolomic and metagenomic approaches can provide a snapshot of the chemical and microbial landscape of an ecosystem.

The metabolomic analyses revealed that the chemical compounds produced by corals, CCA, and macroalgae were statistically different (Fig. 3, Supplementary Data 1). CCA presented the most diverse chemical profile, emphasized by the high percentage of metabolites detected solely in this sample type. In natural systems, CCA colonizes and binds together coral reef substrates. In turn, CCA and its microbial biofilms provide a living and chemically-attractive habitat^63^, that recruits^16^ other reef invertebrates, potentially making their metabolomes and microbiomes complex composites derived from multiple organisms. As expected, the greatest overlap in ion features was between the two algal functional groups we analyzed: CCA and macroalgae. Within coral and macroalgae, chemical profiles varied at the genus level (Fig. 3b, c, e, f), in accordance with previous reports in the literature^37^.

Our results emphasize the potential for metabolomics to be used as a tool for profiling the health of benthic organisms in coral reefs, a priority for the management of these sensitive ecosystems. Concepts that are already familiar to ecologists, like species richness and community dissimilarity, can be helpful when comparing metabolomic profiles across host organisms and geographic locations. Straightforward comparisons of feature richness and sample dissimilarity can reveal broad trends (Fig. 3). In our dataset, it was clear that chemical profiles varied spatially, varying significantly more than the microbiomes did among the same samples. Coral and CCA samples collected immediately adjacent to the Bay (sample code Waimea Bay, Fig. 2) grouped separately from samples collected elsewhere (Supplementary Fig. 3). It is important to note that samples from this site were extracted on a separate 96-well plate, so batch effects may explain some of the observed variation (Supplemental Methods). In terms of habitat, however, the Waimea Bay site was distinct from the other collection sites and we believe this pattern likely represents real biological variation. This site is located near the mouth of the Waimea River, which substantially alters environmental conditions. Within the bay, marine organisms are exposed to periodic runoff, freshwater, and sediment. Corals exposed to these conditions must expend energy to clear colony surfaces, which alters many metabolic processes^64^. In the future, metabolomics may be a sensitive tool for detecting environmental impacts on benthic communities before stressors lead to tissue loss or death.

Of the ion features that were annotated based on spectral matches, most belonged to lipid classes. Lipids are ubiquitous metabolites that perform a variety of cellular functions and are present in both corals and algae^65,66^. In corals, they are an important energy reserve that can represent up to 40% of the coral’s dry mass and are important in supporting physiological resilience and post-bleaching recovery^66^. In algae, lipids play numerous roles in energy storage, membrane formation, and stress response, for which they are considered biomarkers^67^. While lipids are perhaps best known for storing chemical energy and forming bilayer membranes, their emerging role in complex intercellular signaling has become a focus of host-microbe interactions. In heavily studied mammalian systems, short-chain fatty acids are thought to be a key component of crosstalk between the gut microbiome and host organisms^68^. A separate interaction pathway proceeds through the oxidation of membrane phospholipids, which can tip off a host organism to the presence of pathogenic microbes, igniting a signaling cascade of damage control through a process known as ‘innate immunity^69^. Given the importance of oxylipins, in host immune response, it is perhaps unsurprising that microbial symbionts and pathogens are capable of upregulating, modifying, and mimicking them. In corals, it has been established that oxylipins produced by the metazoan host can be modulated by their dinoflagellate endosymbionts, with endosymbionts effectively dampening host immune response^70^. Numerous fungal and bacterial pathogens are capable of dampening host immune response through modification of host oxylipins or through the production of bioactive oxylipins^52^.

Within our dataset, a number of potentially relevant immune signaling compounds were recovered. Glycerophospholipids, including lysophosphatidylcholines (LysoPCs), derivatives such as platelet-activating factor (PAF) C:16, and phosphatidylcholines (PCs) with varied chain extension, were among the most commonly detected lipids in our samples. LysoPCs are widespread in many organisms, including mammals, where they act as proinflammatory signals during immune response. Previous studies have proposed that corals may have an immune system with properties analogous to mammals^37,71^. PAF concentration in *Porites* sp. was observed to increase under stress and inflammatory conditions^37,59^ and has been suggested as a molecular indicator of coral bleaching^36^. In algae, LysoPCs were previously reported in lipidomic analyses of brown algae^72^, and PCs, in general, are common metabolites found in eukaryotic algae and plants. Other lipid classes were also widely detected in our dataset. Fatty acids, which were recovered in abundance, are precursors for the biosynthesis of other lipids^73^, present antibacterial activity against specific pathogenic microorganisms^74^, and are implicated in microbe-host signaling^68^. Several glycerolipids of marine origin have been reported^75^, and acyl carnitines are widely found in corals^31^, being involved in transport across cell membranes^76^ and considered biomarkers of cell toxicity^77^. More specifically, eicosanoids and linoleic acids are reportedly related to immune response in animals, and their biosynthesis was previously related to bacterial infection in corals^37,78^ and insects^79^. Lastly, linoleic acids and their epoxyoctadecamonoenoic acid (EpOMEs) derivatives have been implicated in mouse and lepidopteran immune response to bacteria^80,81^. The potential avenues for lipid mediated host-microbe are both broad and numerous. Through the application of untargeted metabolomics, we were able to identify positive co-occurrence between these putative signaling compounds and microbial taxa with established roles in coral reef ecosystems.

In addition to lipids, our untargeted approach to metabolomics recovered a number of non-lipid compounds. Pigments, such as loliolide and fucoxanthin, were annotated. Fucoxanthin plays an essential role in harvesting light for photosynthesis and photoprotection^82^, while loliolide is an apocarotenoid considered as a photo-oxidative or thermal degradation product of carotenoids^83^. Pheophorbide A, a product of the chlorophyll catabolism, was also observed in macroalgae and CCA samples. Lastly, we recovered a number of purine nucleosides, which are universal molecules with a wide variety of vital biological functions in many organisms, forming building blocks for DNA and RNA. Both macro- and microorganisms in marine environments can produce structurally unusual nucleosides with unique biological properties^84^. There are abundant opportunities to apply untargeted metabolomics to investigate other types of compounds that may be enriched in particular organisms or involved in host-microbe interactions.

A vast majority of molecular features that were statistically associated with different sample types could not be annotated. In fact, the annotation of metabolites in the metabolomics workflow remains a bottleneck in the field. The in silico tools that were employed unquestionably boosted the annotation rates in this dataset, but the annotation of certain metabolites remained intractable. While close to 18% of the metabolites detected in this dataset originated from CCA samples, many features that were primarily detected in CCA, comprising several molecular networks, did not result in any in silico annotations (Supplementary Fig. 5) or library matches. This is consistent with the low annotation rates of exuded metabolites of CCA recently reported^31^. Therefore, CCA represents a source of potentially new compounds to be investigated in future studies. A repository-scale analysis allowed us to determine whether the major networks composed of unannotated CCA MS/MS spectra had been previously reported in other public datasets. Despite comparison to numerous datasets, several CCA associated networks remained unmatched, indicating that CCA might produce distinct molecular families compared to the other reef primary producers, and further emphasizing how little chemical information related to CCA is publicly available. A handful of features from the major CCA molecular family matched with public datasets related to coral reefs, indicating that these compounds are, indeed, found in this environment and are not artifacts. Intriguingly, a molecular family with features matching datasets derived from both coral reefs and cultivated bacteria suggests that some of these compounds may be produced by the microbes associated with CCA. The enigmatic chemical diversity of CCA is noteworthy because of its role in the recruitment of coral larvae. CCA is known to foster coral settlement^16,85^ and suppress the growth of macroalgae^86^, a necessary step for coral reef regeneration. However, the chemical mechanisms by which CCA signals to coral larvae and promotes their growth have not been characterized definitively. There remains much to be discovered about the chemical landscape of these organisms.

Mirroring the metabolomic results, microbial amplicon sequencing revealed that different reef primary producers harbor distinct microbial communities (Fig. 6, Supplementary Data 1). Among coral genera, *Monitipora* and *Porites* presented more similar microbiomes compared with *Pocillopora*, which is in accordance with their closer phylogenetic relatedness^87^. Similarly, the red algae genera *Galaxaura* and *Jania* (Rhodophyta) harbored more similar microbial communities compared to the green alga *Halimeda* (Chlorophyta), further demonstrating that microbial communities tracked the phylogenetic relatedness of the host organisms^88^. Meanwhile, CCA (Rhodophyta, Corallinales) had strikingly different microbiomes compared to the other algal taxa. The microbiomes of CCA were much more similar to those of coral than they were to erect macroalgae, suggesting that the encrusting and calcifying lifestyle of CCA and coral may be an important determinant of microbiome structure. Unlike the metabolomes, which differed by site, the microbial communities were not predictably site-specific (Supplementary Fig. 7). Sampling was conducted during the dry summer months, during which the river mouth was blocked by a sand berm, making it unlikely that substantial inputs of microbes from freshwater were occurring.

A number of microbial taxa were significantly enriched in a particular host (or hosts) and matched previous reports of host-microbe associations^89^. Coral associated microbes included taxa that are thought to be involved in nitrogen recycling in and around the host, which may help corals to persist in oligotrophic waters^90^. Our data indicated several bacterial families associated with nitrogen cycling (Nitrosopumilaceae, Rhizobiales, Nitrosococcaceae, Nitrospiraceae) were associated with coral samples. CCA was associated with many of the same families as corals, including several families involved in nitrogen cycling. Nitrospiraceae are notable for their functional role as nitrite oxidizers while Nitrosopumilaceae are known for their role as ammonia oxidizers^53,54^. Both are thought to contribute to nitrogen cycling in coral holobionts^91^. A prior study reported that Rhizobiales were a core member of CCA microbiomes and that Nitrospiraceae were associated with both CCA and a calcium carbonate substrate control^92^. While Rhizobiales include diazotrophic taxa, their role in nitrogen cycling within the coral microbiome is still unclear^93,94^. Both nitrogen cycling and the presence of calcium carbonate substrates may be key to understanding these host-microbe associations. The family Kiloniellaceae was also highly associated with both CCA and corals; these microbes are putative denitrifiers^95^ and have been reported in association with healthy corals^96^.

There is growing evidence that the association between nitrogen cycling microbes and coral hosts is potentially an important mutualism mediated by chemical exchange of both organic and inorganic forms of nitrogen. Microbial nitrogen transformation, in particular dissimilatory nitrate reduction to ammonium, is thought to be common, though highly variable, in tropical scleractinian corals^97^. Prior work on coral reef exometabolites^31^ showed that coral exudates were enriched in organic nitrogen containing compounds, which were distinct from those exuded by macroalgae, and could potentially drive shifts in water column microbial communities. Many Cyanobacteria are capable of nitrogen fixation and have been identified as important constituents of both coral and macroalgae microbiomes^98,99^. The presence of intercellular cyanobacteria in some coral species indicates that at least some nitrogen-fixing Cyanobacteria are capable of eluding or dampening coral host immune response^99^. In our analysis, macroalgae were associated with several families of Cyanobacteria (Supplementary Fig. 8). Of the prevalent Cyanobacteria in our dataset, most occurred at a higher relative abundance on macroalgae compared to coral and CCA. This trend towards an increased relative abundance of Cyanobacteria on macroalgae merits further investigation.

Macroalgae-associated microbes included copiotrophic taxa (Rhodobacteraceae, Flavobacteriaceae, Vibrionaceae, and Altermonadaceae), which specialize in breaking down large organic molecules and have been reported to increase in response to algal exudates^8^. Previous work has shown that macroalgal exudates are carbon rich, while coral exudates contain higher concentrations of nitrogen and phosphorus^31^. Experimental removal of erect macroalgae from coral in French Polynesia altered the relative abundance of these bacterial families in coral tissues. Both Flavobacteriaceae and Rhodobacteraceae decreased in relative abundance following removal of epiphytic algae from coral^100^. These reports point to a compelling link between increased macroalgae abundance, the release of carbon rich exudates, and increased an abundance of copiotrophic bacteria on both the surface of reef organisms and in the water column. It remains untested whether macroalgae in some way benefit from this association with copiotrophic bacteria, perhaps through increased resource acquisition or competitive advantage.

The observed difference between microbial communities associated with CCA and macroalgae has several possible explanations. CCA harbored a large number of exclusive compounds, any of which could be shaping microbial community structure. Microbiomes could also be responding to the structural differences in surface topology between upright macroalgae and the crustose carbonate substrates characteristic of CCA. Studies testing the effects of surface topology on bacterial settlement have yielded inconsistent findings, but there is evidence that differences in the physical structure of a substrate can impact microbial colonization^101^. The overlap in taxa may have been influenced by our collection method. For CCA and coral, whole cuttings were homogenized and included both surfaces and calcified skeletal components. Skeletal compartments of coral can harbor high microbial diversity and it is possible these microbes contributed to the similarities between these primary producer types^102^. It should be noted that CCA typically harbors a rich community of boring invertebrates, which could have contributed to the metabolic and microbial diversity that we observed in these samples^103^. The large overlap in microbial taxa between CCA and coral is not easily explained, but is notable given the putative role of CCA in coral larval recruitment; both metabolites and microbes derived from CCA are thought to induce the settlement of coral larvae^63^. Microbial families associated with macroalgae were consistent across genera, while microbial families associated with coral varied between the genera *Pocillopora* and *Montipora*/*Porites*. CCA was not identified to the genus level, but the individual samples did not display any clear patterns in microbial associations.

Paired microbe-metabolite datasets are well suited to machine learning techniques, which can identify co-occurrence patterns across a large number of variables. We applied the neural networking tool mmvec^55^ to calculate conditional probabilities of co-occurrence for microbes and metabolites^104–106^. With this multi-omic tool, it was possible to infer possible positive correlations between specific metabolites and microbes, in which a particular microbe was perhaps producing a metabolite, or, vice versa, the presence of a metabolite was inducing the proliferation of a microbe. Beyond these putative direct relationships, co-occurrence patterns could indicate a variety of indirect association between various microbes and metabolites.

Microbes associated with algae co-occurred with a variety of long-chain fatty acids (Fig. 8a). The algal associated microbial families Flavobacteriaceae (Bacteroidia), Saprospiraceae (Bacteroidia), and Rhodobacteraceae (Alphaproteobacteria) all co-occurred with oxidized fatty acid 8-HETE, a compound involved in algal immune response. In contrast to terrestrial plants, which have developed additional advanced anti-microbial response pathways, algae rely heavily on lipid signaling cascades and reactive oxygen species^107^. In two separate studies, 8-HETE was shown to be upregulated after wounding in the red algal species *Gracilaria vermiculophylla*^34^. In a related species, *Gracilaria chilensis*, 8-HETE was upregulated following wounding and, when added directly to *G. chilensis*, decreased settlement by a competing algal epiphyte^108^. In addition to driving microbial community composition through chemical exudates functioning as a food source^31^, perhaps macroalgae are selecting for microbial associates that are tolerant of the oxylipins and reactive oxygen species that are produced during algal immune response.

In the subclass glycerophosphocholines (Fig. 8b) microbe-metabolite co-occurrence was broadly divided between coral associated derivatives of Lyso-PAF and algal associated derivatives of Lyso-PC. Of the 14 compounds, 8 were differentially abundant in one or more primary producer types based on LM analysis. Previous metabolomics studies have identified lyso-PAF as an important compound in coral stress response that can be used to indicate coral health^36,37^. Both coral and algal associated microbes co-occurred with various Lyso-PCs, though algae appeared to harbor a greater number of Lyso-PC related compounds. These compounds are part of lipid synthesis pathways that have multiple connections with symbiosis. Lyso-PC itself has been implicated as a bioactive compound in plants. In plants that form arbuscular mycorrhizal symbioses, the addition of lyso-PC caused rapid alkalinization of plant roots and upregulated transporter genes characteristic of mycorrhizal symbiosis^109^. The base substrate of lyso-PC, phosphatidylcholine (PC), is a common membrane protein in both plants and animals. However, it is uncommon in bacteria and tends to be found in bacteria that have close associations with eukaryotes, including both pathogenic and symbiotic bacteria^110^. When PC synthesis is disrupted in these bacteria, it can result in a loss of virulence in the case of pathogens, and inefficient symbiosis in the case of mutualists^111^. The established role of these glycerophosphocholines in multiple terrestrial symbioses makes them a promising target for further investigation in marine holobiont systems.

Multi-omics approaches to address ecological questions are relatively new, representing an opportunity to explore the impact of microbe-metabolite interactions on ecological systems. Coral reefs are highly complex ecosystems, occurring in a narrow latitudinal margin (~30° N/S) distributed across every continent on the globe apart from Antarctica. Multi-omics tools can shed light both on the commonalities and distinguishing features of these systems. Mmvec is a new tool designed for this type of study. In the Caribbean, a mmvec-based analysis of the coral *Orbicella faveolata* revealed that lipids presented high co-occurrence values with the phylum Firmicutes^106^. In our study, lipids were also key metabolites of reef primary producers, but co-occurred primarily with the phyla Proteobacteria, Bacteroidetes, and Cyanobacteria. Additional comparisons of marine organisms across the globe are likely to reveal distinct and shared co-occurrences patterns.

In conclusion, the present study revealed that, while some ubiquitous features are shared across organisms, different types of coral reef primary producers harbor distinct microbial communities and small molecules. A major pattern in the data was significant site-to-site variation in the metabolomes of each benthic primary producer; in contrast, their microbiomes did not vary significantly between sites. This indicates that benthic primary producers maintain broadly consistent relationships with microbial taxa while their composition of tissue metabolites can be susceptible to change as a result of environmental conditions. Further studies are necessary to identify whether the observed patterns in microbe-metabolite association are driven by interactions that occur intracellularly, on the mucus layers and surface biofilms of tissues, or even within the non-living skeletal components of calcifying taxa. The metabolomics analyses revealed the presence of several biologically relevant lipids, which were primarily detected in coral and macroalgae samples. The in silico annotations allowed us to derive useful information about otherwise unknown metabolites based on structural databases, while multi-omics tools enabled us to investigate biological processes within the reef system. Our results suggest that CCA remains chemically underexplored, representing a relevant target for future studies. The microbiomes of CCA and coral were diverse and overlapped significantly, while the microbiome of macroalgae comprised distinct microbial families. These results provide a comparative view of the complex phylogenetic and chemical contexts in which coral reef symbioses occur. They represent a starting point for future studies to further investigate the complex roles of metabolites, which serve as a nutrient source, signaling language, and physical interface for hosts and microbiomes in holobiont systems.

## Methods

### Sample collection

Samples were collected on June 20th and 21st (2019), from 5 sites in the vicinity of Waimea Bay within the marine life conservation district of Pūpūkea on the north shore of Oʻahu (Fig. 2a). Three biological replicates of each of three species of coral were collected at each site: *Porites lobata*, *Pocillopora meandrina*, and *Montipora capitata* (Fig. 2b–d), resulting in 17, 13, and 15 samples, respectively. Macroalgae were collected in triplicate wherever possible, but not every site had every species in sufficient abundance; we sampled a total of eleven genera: *Colpomenia* (*n* = 2)*, Dictyota* (*n* = 1)*, Ectocarpus* (*n* = 1)*, Galaxaura* (*n* = 5)*, Halimeda* (*n* = 7)*, Hypnea* (*n* = 1)*, Jania* (*n* = 5)*, Lophocladia* (*n* = 1)*, Neomeris* (*n* = 1)*, Porphyra* (*n* = 1) and an unidentified Gigartinales (*n* = 1) (representative Fig. 2e–g). Detailed information on sample size for the collected sample types are described in Supplementary Data 4. Crustose coralline algae were collected as three discrete individuals from each of three morphotypes (examples Fig. 2h–j) at each site as these algae are notoriously difficult to identify, resulting in 41 CCA samples. For coral and CCA samples, divers used stainless steel bone shears to collect ~2 cm^2^ sections of healthy surface material, which included surface biofilms, mucus membranes, living tissue, and calcium carbonate substructures. Entire thalli of macroalgae were cut above the holdfast, which gave individuals on the reef a chance to regenerate post sampling. Samples were collected in clean plastic bags and stored in a cooler on dry ice immediately after collection. Collected samples were rinsed in sterile ultrapure water prior to being frozen and stored at −80 °C. Frozen samples were lyophilized at −50 °C for 48 h (LabConco, Freezone). Entire samples were homogenized post-lyophilization using a Waring blender. The collections were permitted by the Hawaiʻi State Department of Land and Natural Resources Division of Aquatic Resources (Special Activity Permit No. 2020-23).

### DNA extraction and Sequencing analysis

DNA Extractions were conducted on 50 mg (dry weight) subsamples using the PowerSoil MagAttract DNA KF Kit (Qiagen) on a 96-well Kingfisher Flex (Thermofisher Scientific) and a FastPrep-96 homogenizer (MPBio) following the manufacturer’s recommended protocol. A total of 112 samples were subjected to sequencing analyses. The V4 region of the bacterial 16 S gene was amplified in a single step 35 cycle PCR with 12 base pair Golay indexed primers 515 and 806^112^, using the KAPA3G Plant kit (KAPA Biosystems) under the following conditions: 95 °C for 3 min, followed by 35 cycles of 95 °C for 20 s, 50 °C for 15 s, 72 °C for 30 s, and a final extension for 72 °C for 3 min. PCR products were stored at −20 °C prior to downstream cleanup. PCR products were cleaned and normalized using the Just-a-plate kit (Charm Biotech). Normalized PCR products were pooled and concentrated using an SPRI magnetic bead solution (Beckman Coulter). The pooled amplicon library was sequenced using the Illumina Hiseq 2500 platform. Sequencing results were demultiplexed and processed using the MetaFlow|mics custom analysis pipeline^113,114^, which incorporated tools from VSEARCH, Mothur, DADA2, FastTree, and phyloseq. Several filters were imposed throughout the pipeline, as described in the Supplementary Methods. After this pipeline, 93 samples were retained that had >15,000 reads per sample.

### Untargeted MS/MS analysis

The lyophilized materials were weighed and extracted with MeOH:H_2_O (4:1) in a proportion of 100 mg of dried material per 1 mL of extraction solvent. A total of 112 samples were homogenized in a Qiagen TissueLyzer II (Qiagen, Hilden, Germany) for 5 min at 25 MHz and extracted for 30 min at room temperature. The samples were then centrifuged (14,000 *g*) for 15 min in an Eppendorf US centrifuge 5418 (USA), and 600 µL of the supernatants were transferred to a polypropylene 96-deep-well plate. The solvent was evaporated overnight in a Labconco CentriVap (USA), and the plates were sealed and stored at −80 °C until analysis. The dried extracts were initially resuspended in 600 µL MeOH:H_2_O (1:1) containing sulfadimethoxine (1 µM) as an internal standard and sonicated for 15 min. The plates were centrifuged for 10 min at 2000 rpm, and the supernatants were then transferred to a new 96-well plate for metabolomics analyses.

The LC-MS/MS analyses were carried out with a Vanquish UHPLC system coupled to a Q-Exactive Orbitrap mass spectrometer (Thermo Fisher Scientific, Bremen, Germany). In order to minimize batch effects, samples within each plate were randomly injected. For the chromatographic separation, a C18 porous core column (Kinetex C18, 150 × 2 mm, 1.8 µm particle size, 100 A pore size—Phenomenex, Torrance, USA) was used. For gradient elution, a high-pressure binary gradient system was used. The mobile phase consisted of solvent A as H_2_O + 0.1% formic acid (FA), and solvent B as acetonitrile (ACN) + 0.1% FA. The flow rate was set to 0.5 mL/min, injection volume at 5 µL, and the column temperature at 40 °C. After injection, the samples were eluted with the following linear gradient: 0–1 min, 5% B, 1–4 min 5–60% B, 4–10 min 60–99% B, followed by a 3 min washout phase at 99% B and a 3 min re-equilibration phase at 5% B. Data-dependent acquisition (DDA) of MS/MS spectra was performed in positive ionization mode. Electrospray ionization (ESI) parameters were set to 53 AU sheath gas flow, 14 AU auxiliary gas flow, 0 AU sweep gas flow, and 400 °C auxiliary gas temperature; the spray voltage was set to 3.5 kV and the inlet capillary to 320 °C and 50 V S-lens level was applied. MS scan range was set to 150–1500 *m/z* with a resolution at *m/z* 200 (R_*m/z* 200_) of 17,500 with one micro-scan. The maximum ion injection time was set to 100 ms with an automated gain control (AGC) target of 1.0E6. Up to 5 MS/MS spectra per MS^1^ survey scan were recorded in DDA mode with R_*m/z* 200_ of 17,500 with one micro-scan. The maximum ion injection time for MS/MS scans was set to 100 ms with an AGC target of 5E5 ions. The MS/MS precursor isolation window was set to *m/z* 1. The normalized collision energy was set to a stepwise increase from 20 to 30 to 40% with *z* = 1 as default charge state. MS/MS scans were triggered at the apex of chromatographic peaks within 2 to 15 s from their first occurrence. Dynamic precursor exclusion was set to 5 s. Ions with unassigned charge states were excluded from MS/MS acquisition as well as isotope peaks.

### MS/MS data processing and feature-based molecular networking

The raw data files (.raw) were converted to .mzML format using MSconvert (ProteoWizard, Palo Alto, CA, USA)^115^. The .mzML files were processed in MZmine2^116^ (version 2.37.corr17.7_kai_merge2). The parameters used for feature finding are provided in the Supplementary Methods. This feature list was exported as a feature quantification table (.csv), as an MS2 spectral summary (.mgf), and with the SIRIUS export module (.mgf) for downstream analyses.

To investigate and compare the metabolic profile from the CCA, coral, and macroalgae sample types, the processed LC-MS/MS data (.mgf and .csv) were used to create a Feature-Based Molecular Network^43^ on the GNPS platform^46^. Structurally similar compounds present similar fragmentation patterns in mass spectrometry analyses, and the molecular networking approach uses spectral correlation to group ion features with similar structural characteristics. The parameters used for analysis are available in the Supplementary Methods. The metabolites were considered exclusive to a specific producer type if they were not detected in other producers above the threshold used for the MZmine2 data processing. Metabolites for which the maximum peak area in real samples was <3X the maximum peak area in blanks were considered potential contaminants (Supplementary Methods). The molecular networking visualization was performed in Cytoscape (version 3.7.2, Cytoscape consortium, San Diego, CA, USA)^117^.

### Chemical hierarchy analysis

To quantify the chemical hierarchy of the different ion features in the dataset and visualize their distribution across sample types, we used the Qemistree workflow (https://github.com/biocore/q2-qemistree)^44^ available on the GNPS platform^46^. The feature quantification table exported from MZmine2 was used as input, along with the file obtained from the SIRIUS export module (.mgf). More information regarding the steps involved in this analysis can be found in the Supplementary Methods.

### Repository scale analysis

Fragmentation spectra from five entire molecular families (component indexes 10, 42, 71, 184, and 500) containing features mainly detected in CCA samples were submitted to the Mass Spectrometry Search Tool (MASST)^45^, which allows searching a specific MS/MS spectrum in public datasets available in the MassIVE spectral repository. All the MASST jobs in GNPS can be found in Supplementary Data 5.

### Microbe-metabolite associations

To identify associations between microbial taxa and the metabolites they may be producing or consuming, we calculated co-occurrence probabilities. For this analysis, the microbial-metabolite vector (mmvec v1.0.4, https://github.com/biocore/mmvec)^55^ approach was used. Mmvec takes as an input the relative abundance matrices of microbes and metabolites across a shared set of samples. Mmvec then applies a softmax transformation to the data and uses a neural networking approach to determine the conditional probability of observing all metabolites based on the observed abundance of each microbe. A subset of samples with both metabolite and microbiome data were used in this modeling exercise (93 of the 112 samples collected). We selected parameters to optimize a model with low cross-validation error and model likelihood. The parameters chosen were as follows: --p-learning-rate 1e-3, --p-num-testing-examples 30, --p-epochs 200, --p-batch-size 5, --p-latent-dim 3. Emperor^118^ was used to visually inspect the feature-feature biplots. The spheres were colored based on which sample type the metabolites were most abundant, and the arrows indicate the 100 ASVs with the strongest correlations with the PCoA axes.

### Statistics and reproducibility

Statistical analyses were performed to identify the differential enrichment of metabolites and microbes in the three sample types (CCA, coral, and macroalgae). These analyses were performed at several different scales ranging from entire microbial communities and metabolite profiles to individual ion features. At the broadest level, samples’ microbial communities and metabolite profiles were compared using methods based on distance matrices. For metabolites, a Bray-Curtis distance matrix was calculated based on the relative abundance of transformed peak areas. For microbes, a UniFrac distance matrix based on raw read counts was used^119^. Samples were ordinated using non-metric multidimensional scaling (NMDS) as implemented by metaMDS in the R package vegan^120^. Differences in variance and dispersion between sample types, genera, and sampling sites were interrogated using PERMANOVA as implemented in the adonis2 and betadisper functions, also in the R package vegan^120^. To account for potential batch effects, statistical tests were repeated with the extraction plate included as a variable in the model. Though the effect of site on metabolite profiles was diminished, overall trends, in particular the separation between primary producer types, was consistent (Supplementary Methods).

At a more refined level, metabolite networks, metabolite chemical classes, microbial classes, and individual metabolite ion features were tested for differential enrichment in sample types, as described in the Supplementary Methods.

### Reporting summary

Further information on research design is available in the Nature Portfolio Reporting Summary linked to this article.

### Supplementary information

Supplementary Material

Description of Additional Supplementary Files

Supplementary Data 1

Supplementary Data 2

Supplementary Data 3

Supplementary Data 4

Supplementary Data 5

Reporting Summary

## Data Availability

The mass spectrometry data can be accessed on the *Mass spectrometry Interactive Virtual Environment* (MassIVE) at https://massive.ucsd.edu/ as part of the dataset MSV000085129^121^, which is publicly available. The Feature-Based Molecular Networking and the Qemistree jobs can be accessed online at GNPS under the following links: https://gnps.ucsd.edu/ProteoSAFe/status.jsp?task=94d7974737ba4a4c82453f11a3ee1a41 and https://gnps.ucsd.edu/ProteoSAFe/status.jsp?task=55a790571af4490fbf7502d44f65e5c7. All the links for the 158 MASST searches performed can be found in the Supplementary Table 1. Sequence files and sample metadata that support the findings of this study are available from SRA with project number PRJNA701450.

## Code availability

The code and data files used to perform statistical analyses and generate figures for this study can be accessed on Github (https://github.com/soswift/waimea_marine) and in Zenodo^122^.
