# Supplementary material for: Microbiomes and metabolomes of dominant coral reef primary producers illustrate a potential role for immunolipids in marine symbioses
Source: Commun Biol. 2023 Aug 31;6:896. doi: 10.1038/s42003-023-05230-1 (PMC10471604; doi:10.1038/s42003-023-05230-1)
Supplement: Supplementary file 1 — Supplementary Material [file 42003_2023_5230_MOESM1_ESM.pdf]

Supplementary Material

Microbiomes and metabolomes of dominant coral reef primary producers illustrate a potential role for immunolipids in marine symbioses

Helena Mannochio-Russo[1,2,16,*], Sean O. I. Swift[3,16,*], Kirsten Nakayama[4], Christopher B. Wall[4,5], Emily C. Gentry[1,6], Morgan Panitchpakdi[1,6], Andrés M. Caraballo-Rodriguez[1], Allegra T. Aron[1,7], Daniel Petras[1,8], Kathleen Dorrestein[1,6], Tatiana Dorrestein[9], Taylor M. Williams[10], Eileen M. Nalley[11], Noam T. Altman-Kurosaki[12], Mike Martinelli[13], Jeff Kuwabara[10], John L. Darcy[4], Vanderlan S. Bolzani[2], Linda Wegley Kelly[14], Camilo Mora[15], Joanne Y. Yew[4], Anthony S. Amend[4], Margaret McFall-Ngai[4], Nicole A. Hynson[4], Pieter C. Dorrestein[1,6], Craig E. Nelson[3]

[1]Skaggs School of Pharmacy and Pharmaceutical Sciences, University of California, San Diego, La Jolla, CA 92093, USA.

[2]Department of Biochemistry and Organic Chemistry, Institute of Chemistry, São Paulo State University, Araraquara, SP 14800-060, Brazil.

[3]Daniel K. Inouye Center for Microbial Oceanography: Research and Education, Department of Oceanography and Sea Grant College Program, University of Hawaiʻi at Mānoa, Honolulu, HI 96822, USA.

[4]Pacific Biosciences Research Center, University of Hawaiʻi at Mānoa, Honolulu, HI 96822, USA.

[5]Ecology Behavior and Evolution Section, Department of Biological Sciences, University of California, San Diego, La Jolla, CA 92093, USA.

[6]Collaborative Mass Spectrometry Innovation Center, Skaggs School of Pharmacy and Pharmaceutical Sciences, University of California, San Diego, La Jolla, CA 92093, USA.

[7]Department of Chemistry and Biochemistry, University of Denver, Denver, CO, 80210, USA.

[8]Cluster of Excellence "Controlling Microbes to Fight Infections" (CMFI), University of Tuebingen, Tuebingen, Germany.

[9]University City High School, San Diego, CA 92122, USA.

[10]Marine Option Program, University of Hawaiʻi at Mānoa, Honolulu, HI 96822, USA.

[11]Hawaiʻi Sea Grant College Program, University of Hawaiʻi at Mānoa, Honolulu HI 96822 USA.
[12]School of Biological Sciences, Georgia Institute of Technology, 311 Ferst Drive, Atlanta, GA, 30332, USA.
[13]University of Hawaiʻi at Mānoa, Honolulu, HI 96822, USA.
[14]Scripps Institution of Oceanography, University of California San Diego, La Jolla, California, USA.
[15]Geography, University of Hawaiʻi at Mānoa, Honolulu, HI 96822, USA.
[16]These authors contributed equally.

Corresponding authors:
Helena Mannochio-Russo; helenamrusso@gmail.com
Sean O. I. Swift; seanoiswift@gmail.com

Summary

Supplementary Methods **5**

Supplementary Note 1 **11**

Supplementary Figure 1. Non-metric Multidimensional Scaling plot of metabolites by primary producer type using Bray-Curtis distance metric produced from 11,215 ion features (acquired from 112 samples) relative abundances, considering the blank analyses. **13**

Supplementary Figure 2. Non-metric Multidimensional Scaling plot of metabolites by primary producer type using Bray-Curtis distance metric produced from 8,221 ion features relative abundances, considering the blank analyses. **13**

Supplementary Figure 3. Non-metric Multidimensional Scaling plot of metabolites by site within each primary producer type. Bray-Curtis distance metric produced from 8054 ion feature relative abundances was employed. Panels show the collection sites in (A) macroalgae, (B) coral, and (C) CCA samples. **14**

Supplementary Figure 4. Molecular networks obtained for the coral reef primary producer types in this study: CCA (blue), coral (orange), and macroalgae (green). Node sizes are relative to the sum of the precursor ion intensity in MS1 scans. Nodes with matches to the GNPS libraries are shown in a rounded squared shape. **15**

Supplementary Figure 5. Major networks composed of metabolites mainly detected in CCA samples, which did not present any library match. Node sizes are relative to the summed peak areas of the precursor ion in MS1 scans. Information regarding the significance of each feature in Random Forest and Linear Model algorithms is shown. Component indexes of each network are depicted. **16**

Supplementary Figure 6. Dendrograms obtained from the Qemistree workflow. Trees were pruned to keep only fingerprints classified up to a superclass, class, and subclass level (Classyfire ontology). Legends show the most abundant classifications obtained. **17**

Supplementary Figure 7. Non-metric Multidimensional Scaling plot of microbes by site within each primary producer type. Unifrac distance metric produced from 36,009 ASV relative abundances was employed. Panels show the collection sites in (A) macroalgae, (B) coral, and (C) CCA samples. **17**

Supplementary Figure 8. Violin plots of log relative abundance for microbial families in class Cyanobacteriia. Only families that were present in more than half the samples are shown. The subtitle indicates whether the microbial family was determined to be differentially abundant in a particular primary producer type. **18**

Supplementary Figure 9. Additional mmvec ordinations. Ellipses indicate 95% confidence intervals for metabolites that were enriched in the three sample types. Arrows indicate microbial ASVs belonging to specific families. Arrows pointing towards a highlighted sample type region indicate microbes co-occurring with metabolites enriched in that sample type. **19**

Supplementary Figure 10. Biclustering analysis of the multi-omics data. Chemical hierarchy obtained from the Qemistree workflow is represented in Y-axis, while the microbial 16S phylogeny is shown in X-axis. The heatmap indicates the co-occurrences probabilities calculated by mmvec. Summed relative abundance of the metabolites and microbes in each marine organism were added as bar plots. Microbial taxonomic classification (class and order) and

chemical structural classification (direct parent and network) was added. **20**
Supplementary References **21**

Supplementary Methods

Sequencing analysis

Sequencing results were demultiplexed and processed using the MetaFlow|mics custom analysis pipeline[1,2], which incorporated tools from VSEARCH, Mothur, DADA2, FastTree, and phyloseq. Several filters were imposed throughout the pipeline. Only reads longer than 20 bp were retained. Forward and reverse reads were truncated at 250 bp and 220 bp or at the first base with quality lower than 2. Reads with more than 3 expected errors were discarded. Similarly, paired reads that overlapped by less than 20 bp or had greater than one mismatch in the merge region were discarded. No sequence identity clustering was performed so that bacterial taxa were identified as amplicon sequence variants (ASVs). Taxonomic assignments were performed against the Silva database (version 138)[3], with a minimum alignment length of 50 bp. Sequences were additionally filtered by taxonomic criteria: taxa matching mitochondria and chloroplasts were discarded from subsequent analyses, as were those that could not be assigned to a Domain. Finally, uninformative sequences were discarded based on low occurrence in the dataset: ASVs retained had to be observed at an abundance of 0.001% in at least 3 samples or at an abundance of 0.1 % in a single sample. Finally, to standardize sequencing effort, reads were randomly subsampled to 15,000 reads per sample.

Quality control and quality assurance for metabolomics and DNA sequencing

The metabolomics solvent extractions were conducted in 96-well plates. A blank containing the resuspension solvent (MeOH:H_2O (1:1) containing 1 µM sulfadimethoxine) and a quality control containing a mixture of six standards was injected every 8 samples. The quality of the analyses was evaluated considering the retention time and the *m/z* of this mixture of standards (sulfamethizole, sulfamethazine, sulfachloropyridazine, sulfadimethoxine, amitryptiline, and coumarin). After MS/MS data processing, the features that presented a ratio of blank/sample abundance of > 1:3 were inspected and considered as possible contaminants (**Supplementary Data 2**). Solvent extractions were spread across two 96-well plates. The first plate contained a majority of the samples, while the second plate comprised all of the samples from Waimea Bay (N=18) and ~¼ of the samples from Sharks Cove (N=4). Because of the Waimea Bay site's proximity to the river mouth, these samples were expected to vary substantially from the other

samples in the dataset. However, to ensure that batch effects were not responsible for the broad trends observed in the data, a PERMANOVA model was constructed with extraction plate included as an additional predictive variable (formula: Bray-Curtis Distance ~ Extraction Plate + Benthic Primary Producer Type * Site). Primary producer type remained the largest predictor of metabolite profiles ($R^2 = 0.15$; $p = 0.001$). The interaction between sample type and collection site likewise remained largely unchanged ($R^2 = 0.08$; $p = 0.001$). Differences between collection sites remained significant, though, as expected, they explained less variation than when inter plate variation was not accounted for ($R^2 = 0.04$; $p = 0.003$). The overall trends in the data, in particular the distinct metabolite profiles presented by the different primary producer types, remained consistent whether or not potential batch effects were accounted for. In our analysis, we elected to interpret the variation between samples, particularly the relatively distinct samples from Waimea Bay, as real biological variation rather than batch effects.

For DNA sequencing, samples were randomized across extraction and PCR plates. DNA extractions were performed as part of a larger set of samples and were spread across multiple 96-well extraction plates (N =11). A PCR negative and a DNA extraction negative from each plate were sequenced alongside the samples. After running both real samples and controls through the processing pipeline, sequences were clustered at 97% similarity to aid in the identification of potential contaminants. The total read counts and total number of unique OTUs were compared across all samples that were included in our final analysis (N = 93), all extraction negatives (N = 11), and all PCR negatives (N = 11). Mean read counts and OTU counts were as follows: extraction negatives (Avg. Reads = 35,063; Avg. OTUs = 56), PCR negatives (Avg. Reads = 27,809, Avg. OTUs = 30), real samples (Avg. Reads = 78,491, Avg. OTUs = 889). Real samples generated more sequences than the negative controls, and the biological diversity of real samples was substantially higher than the negative controls. Dominant taxa in the negative controls included known reagent contaminants associated with freshwater or the human microbiome and did indicate cross-contamination between samples. The top genera associated with negative controls included *Ralstonia*, *Asinibacterium*, *Burkholderia*, *Corynebacterium*, and *Bradhyrhizobium*. The overall impact of microbial contaminants on our analyses was deemed to be minimal.

MS/MS data processing

The parameters used for feature finding were as follows: mass detection (centroid, 1.0E5 and 1.0E3 for MS1 and MS2, respectively); ADAP chromatogram builder[4] (minimum group size in scan set to 5, group intensity threshold of 1.0E5, minimum highest intensity of 3.0E5 and *m/z* tolerance of 0.001 *m/z* or 20 ppm); chromatogram deconvolution (local minimum search algorithm: chromatographic threshold of 0.1%, a search minimum in retention time [RT] range of 0.2 min, minimum relative height of 1%, the minimum absolute height of 1.0E5, a minimum ratio of peak top/edge set to 1, and peak duration range as 0.01 to 1.5 min) with median *m/z* center calculation, *m/z* range for MS2 scan pairing of 0.01 Da and RT range for MS2 scan pairing of 0.1 min; isotope peaks grouper (*m/z* tolerance set at 0.001 or 10 ppm, RT tolerance of 0.2 min, maximum charge of 3, and representative isotope set to the most intense), join alignment (*m/z* tolerance of 0.001 *m/z* or 10 ppm, weight for *m/z* and RT of 90 and 10, respectively, and RT tolerance of 0.2 min). The last step consisted of applying filters in which only the features that had MS/MS spectra eluting from 0 to 10 min that were present in at least 3 samples were considered.

Feature-Based Molecular Networking

The data were filtered by removing all MS/MS fragment ions within +/- 17 Da of the precursor ion. Additionally, MS/MS spectra were window filtered to select only the top 6 fragment ions in the +/- 50 Da window throughout the spectrum. Both the precursor ion and the MS/MS fragment ion tolerance were set to 0.02 Da. A molecular network was created where edges were filtered to have a cosine score above 0.7 and more than 4 matched peaks. Similarly, the parameters for the library search (for comparison between the experimental and library spectra) were set to have a score above 0.7 and at least 4 matched peaks to assist in the metabolites' annotation - levels two or three according to the metabolomics standards initiative[5]. We searched the experimental data in the public spectral database Speclibs available in GNPS.

Cytoscape molecular networking visualization

Molecular network visualization was performed in Cytoscape (version 3.7.2, Cytoscape consortium, San Diego, CA, USA)[6], in which the nodes correspond to the MS1 precursor ion features, and the edges represent the MS/MS cosine scores calculated between two nodes.

Molecular families containing features detected in blank samples (i.e. more than half of the relative abundance based on peak area) were excluded from the Cytoscape visualization to avoid misinterpretations due to contaminants. Sample type information was added to color the nodes as pie charts representing the relative abundance of the features across the samples (coral colored as orange, CCA as blue, and macroalgae as green). Node size is scaled relative to the sum of the peak areas obtained in the samples in which the feature was detected. Compounds with the same MS/MS spectra, but with different retention times, are represented as separate nodes, indicating isomers.

Chemical hierarchy analysis

To quantify the chemical hierarchy of the different ion features in the dataset and visualize their distribution across sample types, we used the Qemistree workflow (https://github.com/biocore/q2-qemistree)[7] available on the GNPS platform[8]. The feature quantification table exported from MZmine2 was used as input, along with the file obtained from the SIRIUS export module (.mgf). In summary, the Qemistree workflow consists of applying SIRIUS[9] to the feature table, which generates information regarding the predicted molecular formulas of each metabolite by estimating a fragmentation tree that best explains the observed fragmentation spectrum. The predicted molecular formulas were reranked using ZODIAC[10], the predicted molecular fingerprints were subsequently generated using fragmentation trees via CSI:FingerID[11], and the chemical taxonomy of the predicted metabolite structures was obtained by ClassyFire[12] (kingdom, superclass, class, subclass, and direct parent). The Euclidean pairwise distances between these molecular fingerprints were calculated, and the fingerprint vectors were hierarchically clustered using the unweighted pair group method with arithmetic mean to generate a tree that represents the structural chemical relationships of this dataset. This tree was visualized interactively in iTOL[13] for data exploration. Sample type information was added as relative abundance stacked bar charts for each feature and the main predicted classes of compounds were shown as pie charts on internal tree nodes. The Qemistree job on GNPS can be found at https://gnps.ucsd.edu/ProteoSAFe/status.jsp?task=55a790571af4490fbf7502d44f65e5c7.

Repository scale analysis

Fragmentation spectra from entire molecular families containing features mainly detected in CCA samples were submitted to the Mass Spectrometry Search Tool (MASST)[14], which allows searching a specific MS/MS spectrum in public datasets available in the MassIVE spectral repository. Analogously to the FBMN job, the input and library data were filtered by removing all MS/MS fragment ions within +/- 17 Da of the precursor *m/z*, and the MS/MS spectra were window filtered by choosing only the top 6 fragment ions in the +/- 50 Da window throughout the spectrum. Both the precursor ion mass tolerance and the precursor ion mass tolerance were set to 0.01 Da. The matches between the input spectra and the library spectra were required to have a cosine score above 0.7 and a minimum of 6 matched peaks. The MASST jobs in GNPS can be found in Supplementary Table 1.

Statistical analysis

The metabolite networks, metabolite chemical classes, microbial classes, and individual metabolite ion features were tested for differential enrichment in sample types. The general methodology was to sum relative abundance for the selected group (e.g. network, class, etc.); normalize the data through arc-sine square root transformation; and finally test for the effect of sample type using a simple anova. To account for multiple testing, P-values derived from these anovas were adjusted using the Benjamini-Hochberg method. A post-hoc Tukey's test was used to test for pairwise differences between the three sample types. The log2 fold change in mean relative abundance between sample types was calculated to provide information on the magnitude of differential enrichment.

Individual metabolite ion features were analyzed using a methodology similar to the one described above for classes and networks. In the case of individual metabolites, relative abundance was transformed using the centered log-ratio method rather than arc-sine square root. Apart from that, linear methods followed those described above. In addition to traditional statistical methods, a random forest analysis was used to identify 'important' features that could be used to predict sample type. The function randomForest, as implemented in the R package randomForest, was run on the entire dataset with the number of trees set to 500. The mean decrease in accuracy over all classes was used to assess ion feature importance.

Biclustering analysis

The biclustering analysis was performed based on the results of mmvec, Qemistree, and phylogenetic analysis of the microbial sequences. Microbial taxa were organized phylogenetically using a 16S tree assembled with FastTree[15]. Metabolites were organized by structural similarity using the tree output from Qemistree. Metabolite probability scores were standardized for each metabolite through a z-score transformation so that uniform cutoff values could be applied across metabolites.

In our study, samples were collected from 3 distinct types of host organisms. Host organisms were likely the source of a significant portion of the observed metabolites. The coloring scheme for the bicluster highlighted cases in which a metabolite and a microbe were 1) associated with each other; and 2) associated with the same sample type. Each association in the bicluster was filled if the z-scored probability was greater than one, indicating a co-occurrence probability at least one standard deviation higher than the mean for that metabolite. The association was colored if the microbe and metabolite both had the highest mean abundance in the same sample type. Conversely, if the microbe and metabolite occurred abundantly in different sample types, the association was colored gray (Not Same). Finally, in order to reduce noise, only metabolites that had a median mmvec score >= 2 were retained. After filtering, 1,266 microbes and 438 metabolites features were retained, as shown in Supplementary Fig. 10.

Supplementary Note 1

Inspection of the spectral matches statistically significant retrieved from the Random Forest and Linear Models.

Spectral matches to glycerophospholipids were among the most observed within the criteria defined (Fig. 4a), particularly abundant in coral and macroalgae. These spectral matches are level 2 annotations according to the 2007 Metabolomics Standard Initiative[5], and the compounds present in the samples could be their isomers. Among the networks and library matches shown, five features stood out for being statistically significant in both RF and LM: 1-hexadecyl-sn-glycero-3-phosphocholine (**1901**), lyso-PAF C-18 (**25755**), 1-stearoyl-2-hydroxy-sn-glycero-3-phosphocholine (**24792** and **7266**), and lyso-PC(16:0) (**166**). These features were correlated to coral in the LM and showed to be differentially abundant for this sample type, except for feature **166**, which showed to be differentially abundant for both coral and macroalgae. Other features with spectral matches to glycerophospholipids were also highlighted only in LM, such as 1-(1Z-hexadecenyl)-sn-glycero-3-phosphocholine (**25753**), PAF C-16 (**37817**), 18:1 lyso PC (**5327**), 1-(1Z-hexadecenyl)-sn-glycero-3-phosphocholine (**37250**), 1-hexadecanoyl-sn-glycero-3-phosphocholine (**978**), and 1-(9Z-octadecenoyl)-sn-glycero-3-phosphoethanolamine (**25921**). Features **37817**, **37250**, and **25921** also showed differential abundance in coral, while feature **5327** was differentially abundant in macroalgae, and **978** in both coral and macroalgae.

Several networks containing spectral matches with fatty acids and derivatives were also highlighted in RF, LM, or both. Pinolenic acid (**9906**) and 2,4-dihydroxyheptadecyl acetate (**453**) were highlighted in both statistical treatments, showing differential abundance related to coral, and both coral and macroalgae, respectively. Spectral matches to 19(20)-EpDPE (**25194**, **32167**, **15247**, and **15781**), 8-HETE (**16992**), and cis-8,11,14-eicosatrienoic acid (**1450**) were only retrieved from LM, showing to be differentially abundant in corals. Similarly, 17(18)-EpETE (**5263**) and 9(10)-EpOME (**2801**) were differentially abundant in macroalgae, and 13-keto-9,11-octadecadienoic acid (**8476**) was retrieved from the RF algorithm.

A network containing spectral matches to acylcarnitines was also obtained, with library matches to propionylcarnitine (**30486**), valerylcarnitine (**26352**), and palmitoylcarnitine (**36640**). All of these features showed to be differentially abundant in corals in the LM evaluated. In addition, two networks related to glycerolipids were observed, matching 9,12,15-octadecatrienoic acid 3-(hexopyranosyloxy)-2-hydroxypropyl ester (**15641**), 1-monopalmitolein (**1200**, **667**, **6983**), and monoelaidin (**1691**). From these features, **1200** was retrieved in both RF and LM (differentially abundant in macroalgae), while **15641** and **1691** were only retrieved from LM, with differential abundance in coral and macroalgae, respectively.

Terpenoids were also detected in addition to the lipid-like molecules (Fig. 4b). A library match to fucoxanthin (**127**) carotenoid was obtained and showed to be differentially abundant in macroalgae samples according to the LM. The library searches also retrieved loliolide (an apocarotenoid, **524**), classified as significant in both RF and LM, being differentially abundant in macroalgae. In addition, the LM also retrieved bisabolol (feature **14018**) as being differentially abundant in macroalgae samples.

Several matches to purine nucleosides (Fig. 4c) were also observed, in which 2'-deoxyadenosine (**423**) was differentially abundant in coral samples by the LM, besides being labeled as an important feature in RF. According to the LM, adenosine (**42529**) was differentially abundant in both coral and CCA samples. A small network was also observed, containing library matches to pheophorbide A (Fig. 4d) (**404**), a product from the chlorophyll catabolism, which was statistically significant both in RF and LM, being differentially abundant in CCA. Lastly, a network containing matches to phthalates (Fig. 4e) was also observed, with dibutyl phthalate (**7611**) being differentially abundant in macroalgae samples according to the LM.

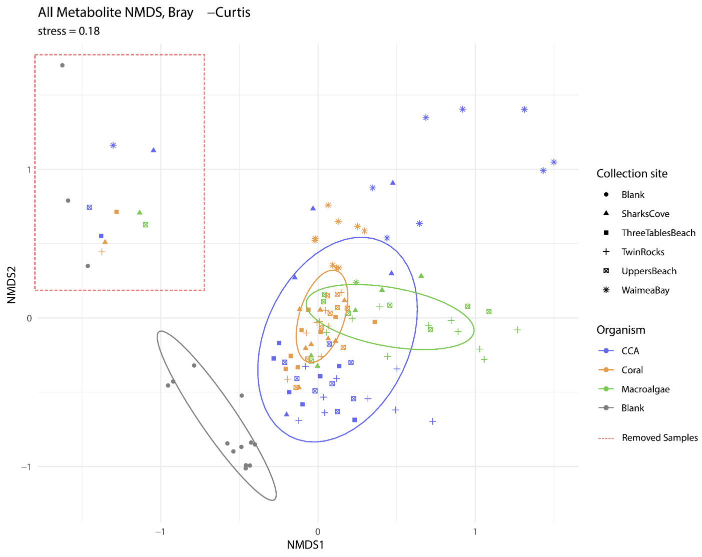

Supplementary Figure 1. Non-metric Multidimensional Scaling plot of metabolites by primary producer type using Bray-Curtis distance metric produced from 11,215 ion features (acquired from 112 samples) relative abundances, considering the blank analyses.

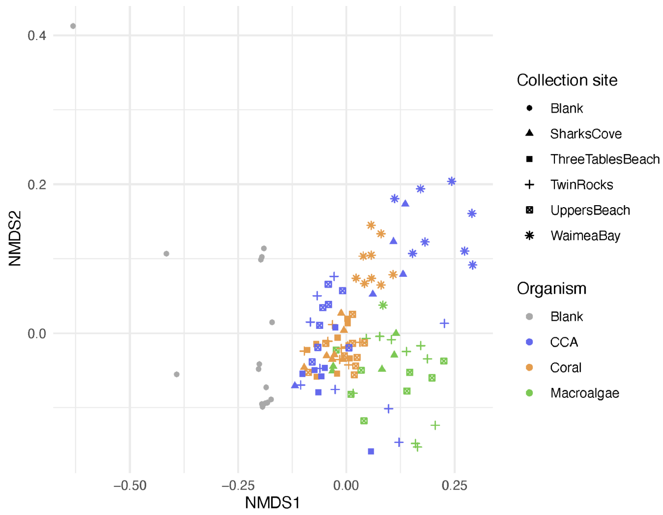

Supplementary Figure 2. Non-metric Multidimensional Scaling plot of metabolites by primary producer type using Bray-Curtis distance metric produced from 8,221 ion features relative abundances, considering the blank analyses.

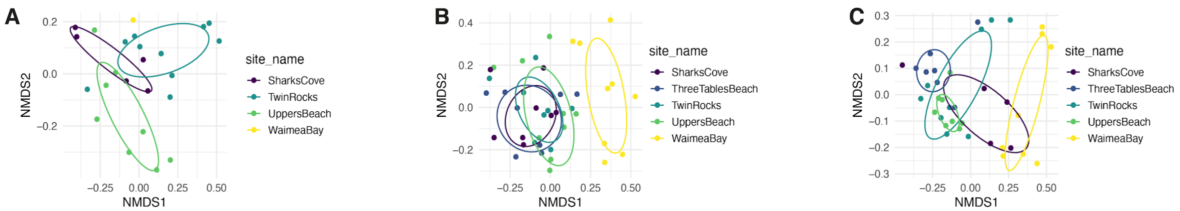

Supplementary Figure 3. Non-metric Multidimensional Scaling plot of metabolites by site within each primary producer type. Bray-Curtis distance metric produced from 8054 ion feature relative abundances was employed. Panels show the collection sites in (A) macroalgae, (B) coral, and (C) CCA samples.

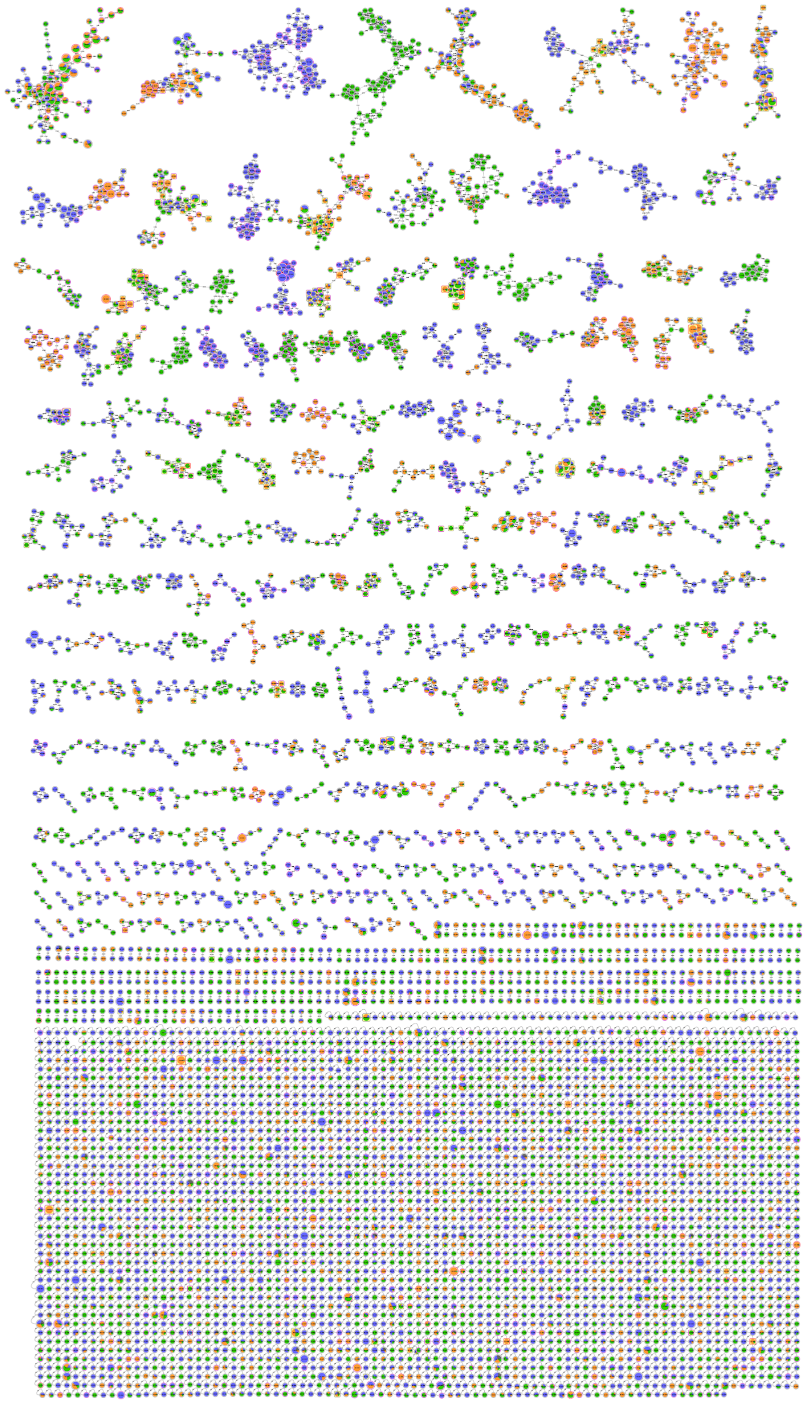

Supplementary Figure 4. Molecular networks obtained for the coral reef primary producer types in this study: CCA (blue), coral (orange), and macroalgae (green). Node sizes are relative to the sum of the precursor ion intensity in MS1 scans. Nodes with matches to the GNPS libraries are shown in a rounded squared shape.

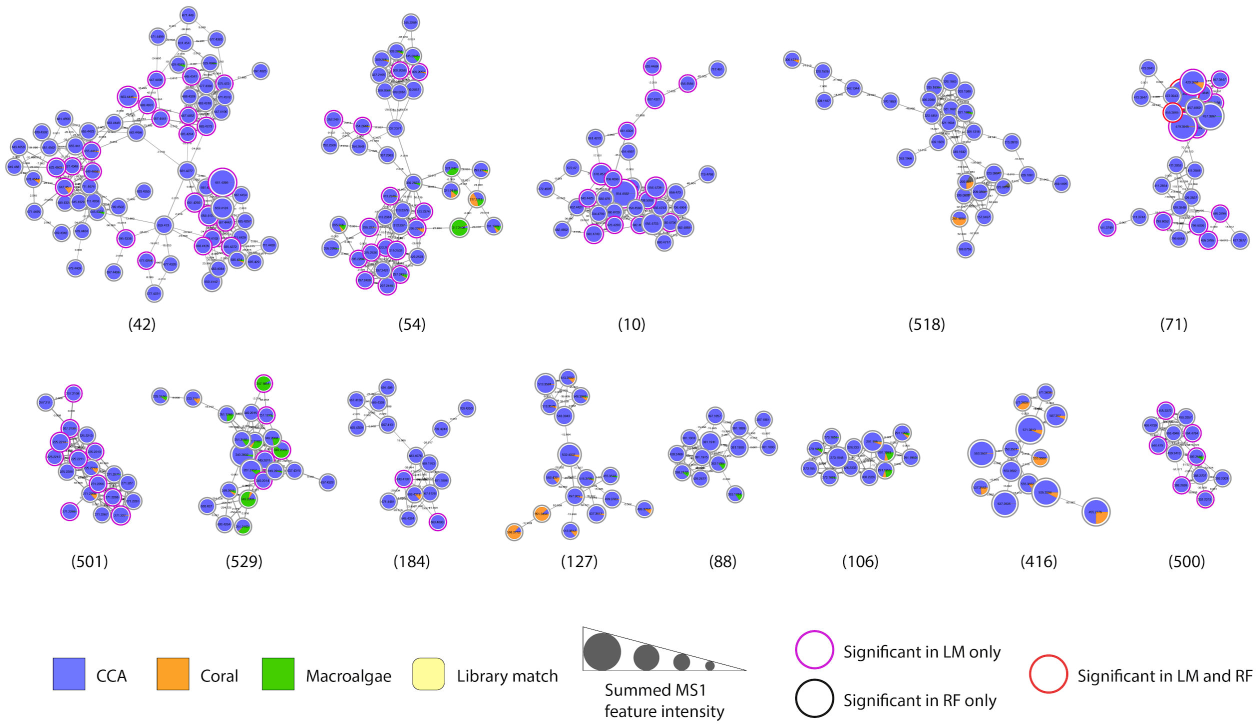

Supplementary Figure 5. Major networks composed of metabolites mainly detected in CCA samples, which did not present any library match. Node sizes are relative to the summed peak areas of the precursor ion in MS1 scans. Information regarding the significance of each feature in Random Forest and Linear Model algorithms is shown. Component indexes of each network are depicted.

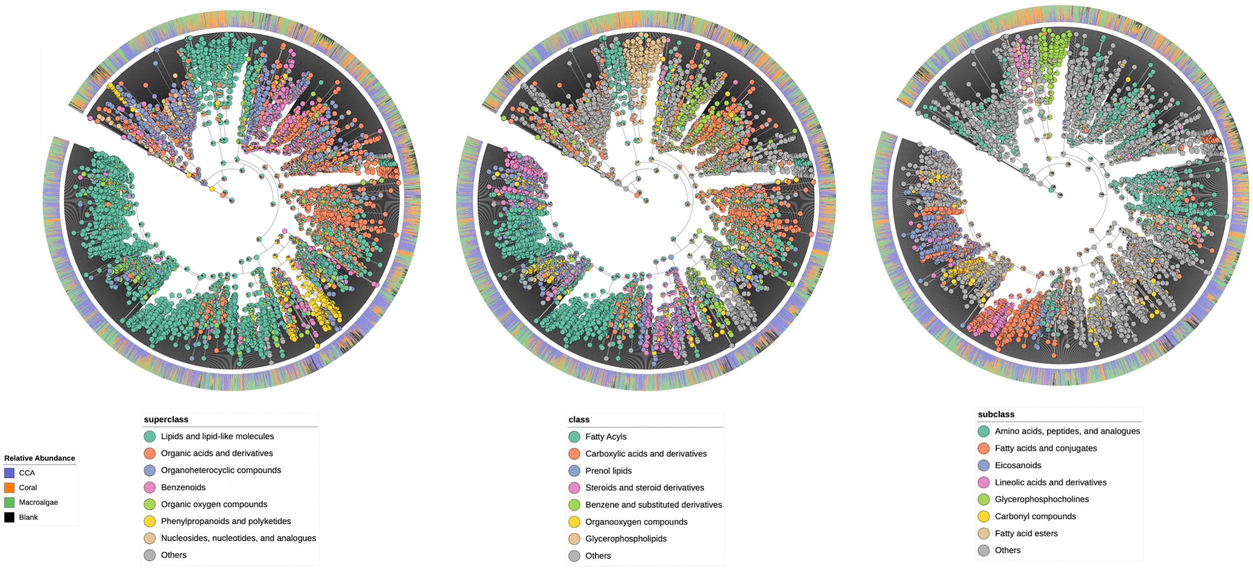

Supplementary Figure 6. Dendrograms obtained from the Qemistree workflow. Trees were pruned to keep only fingerprints classified up to a superclass, class, and subclass level (Classyfire ontology). Legends show the most abundant classifications obtained.

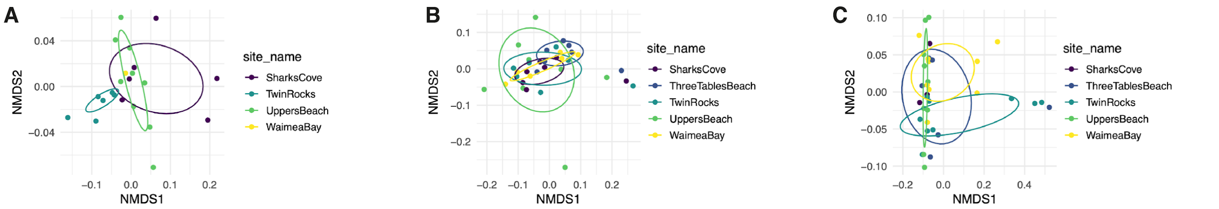

Supplementary Figure 7. Non-metric Multidimensional Scaling plot of microbes by site within each primary producer type. Unifrac distance metric produced from 36,009 ASV relative abundances was employed. Panels show the collection sites in (A) macroalgae, (B) coral, and (C) CCA samples.

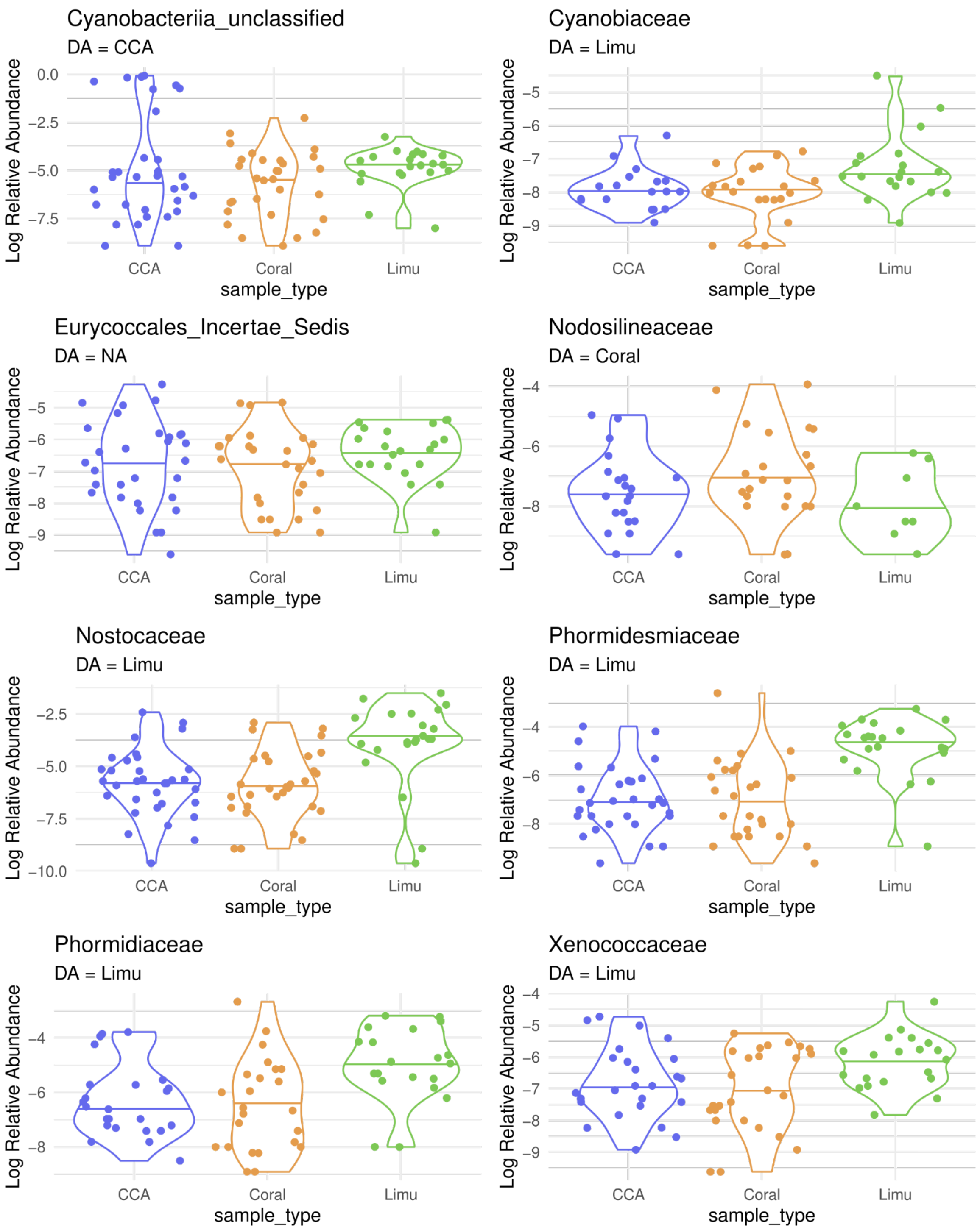

Supplementary Figure 8. Violin plots of log relative abundance for microbial families in class Cyanobacteriia. Only families that were present in more than half the samples are shown. The subtitle indicates whether the microbial family was determined to be differentially abundant in a particular primary producer type.

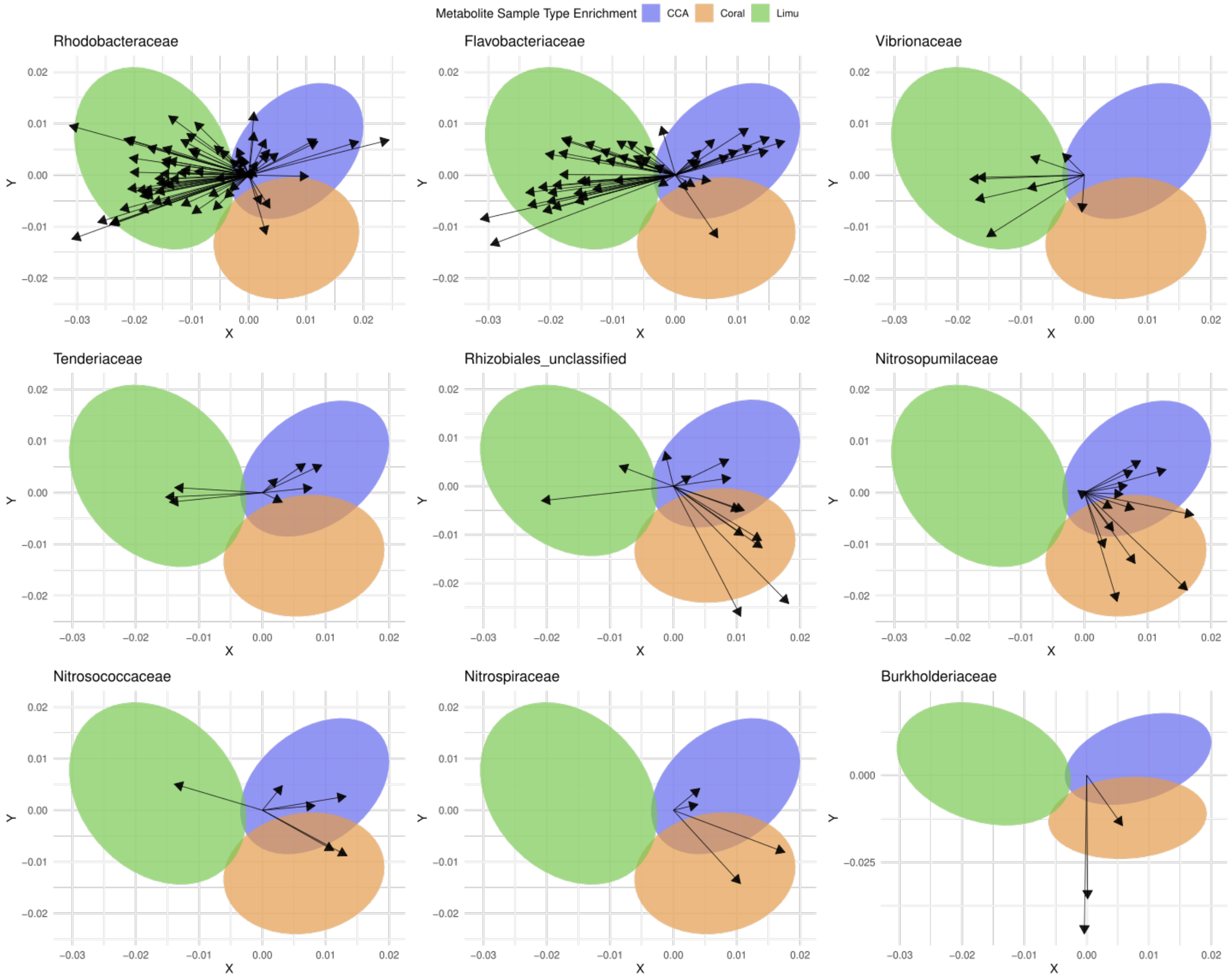

Supplementary Figure 9. Additional mmvec ordinations. Ellipses indicate 95% confidence intervals for metabolites that were enriched in the three sample types. Arrows indicate microbial ASVs belonging to specific families. Arrows pointing towards a highlighted sample type region indicate microbes co-occurring with metabolites enriched in that sample type.

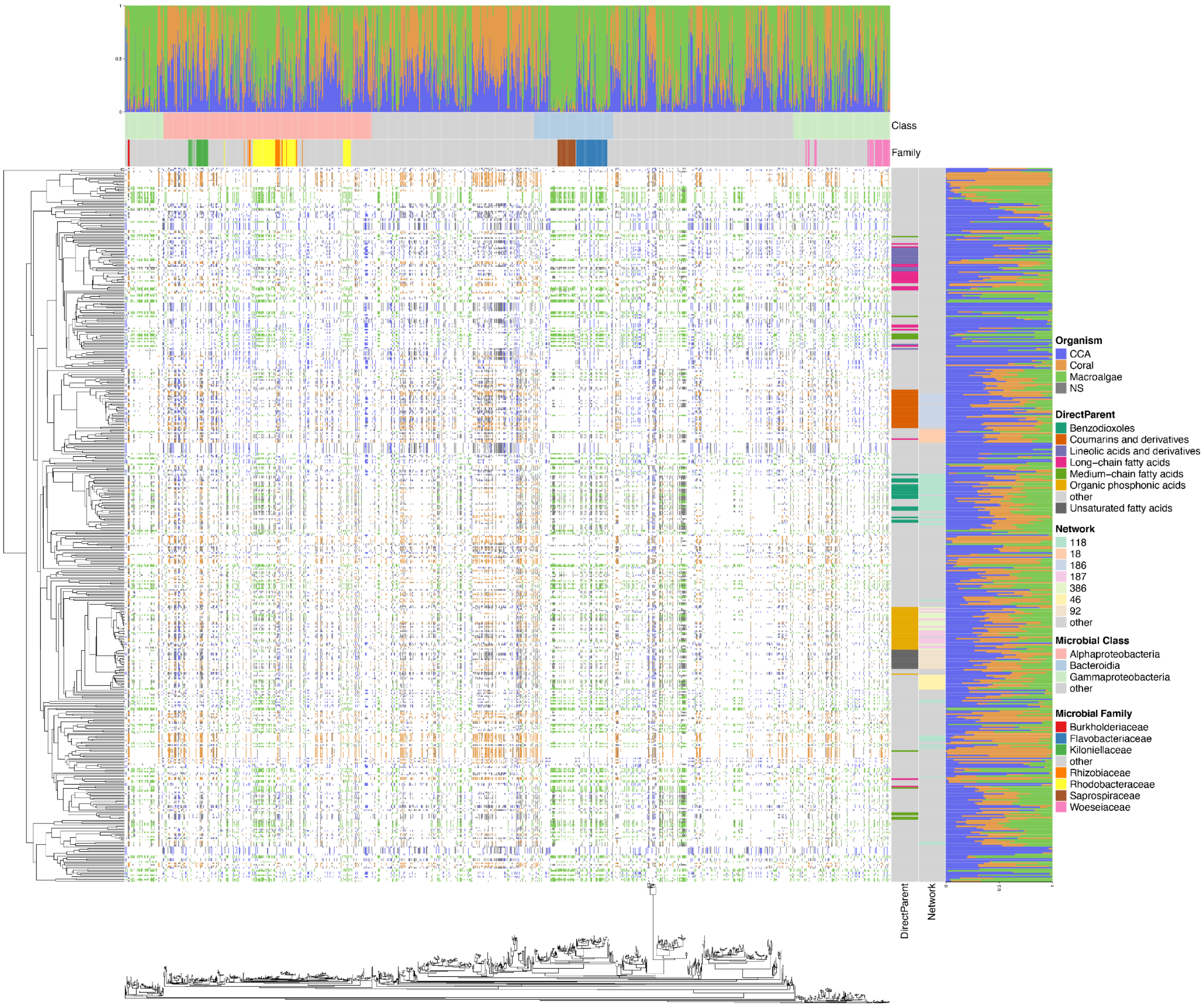

Supplementary Figure 10. Biclustering analysis of the multi-omics data. Chemical hierarchy obtained from the Qemistree workflow is represented in Y-axis, while the microbial 16S phylogeny is shown in X-axis. The heatmap indicates the co-occurrences probabilities calculated by mmvec. Summed relative abundance of the metabolites and microbes in each marine organism were added as bar plots. Microbial taxonomic classification (class and order) and chemical structural classification (direct parent and network) was added.

Supplementary References

1. Jani, A. J. *et al.* The amphibian microbiome exhibits poor resilience following pathogen-induced disturbance. *ISME J.* (2021) doi:10.1038/s41396-020-00875-w.
2. Arisdakessian, C., Cleveland, S. B. & Belcaid, M. MetaFlow|mics: Scalable and reproducible nextflow pipelines for the analysis of microbiome marker data. in *Practice and Experience in Advanced Research Computing* (ACM, 2020). doi:10.1145/3311790.3396664.
3. Quast, C. *et al.* The SILVA ribosomal RNA gene database project: improved data processing and web-based tools. *Nucleic Acids Res.* **41**, D590–6 (2013).
4. Myers, O. D., Sumner, S. J., Li, S., Barnes, S. & Du, X. One Step Forward for Reducing False Positive and False Negative Compound Identifications from Mass Spectrometry Metabolomics Data: New Algorithms for Constructing Extracted Ion Chromatograms and Detecting Chromatographic Peaks. *Anal. Chem.* **89**, 8696–8703 (2017).
5. Sumner, L. W. *et al.* Proposed minimum reporting standards for chemical analysis Chemical Analysis Working Group (CAWG) Metabolomics Standards Initiative (MSI). *Metabolomics* **3**, 211–221 (2007).
6. Shannon, P. *et al.* Cytoscape: a software environment for integrated models of biomolecular interaction networks. *Genome Res.* **13**, 2498–2504 (2003).
7. Tripathi, A. *et al.* Chemically informed analyses of metabolomics mass spectrometry data with Qemistree. *Nat. Chem. Biol.* **17**, 146–151 (2021).
8. Wang, M. *et al.* Sharing and community curation of mass spectrometry data with Global Natural Products Social Molecular Networking. *Nat. Biotechnol.* **34**, 828–837 (2016).
9. Dührkop, K. *et al.* SIRIUS 4: a rapid tool for turning tandem mass spectra into metabolite

structure information. *Nat. Methods* **16**, 299–302 (2019).

10. Ludwig, M. *et al.* Database-independent molecular formula annotation using Gibbs sampling through ZODIAC. *Nature Machine Intelligence* **2**, 629–641 (2020).
11. Dührkop, K., Shen, H., Meusel, M., Rousu, J. & Böcker, S. Searching molecular structure databases with tandem mass spectra using CSI:FingerID. *Proc. Natl. Acad. Sci. U. S. A.* **112**, 12580–12585 (2015).
12. Djoumbou Feunang, Y. *et al.* ClassyFire: automated chemical classification with a comprehensive, computable taxonomy. *J. Cheminform.* **8**, 61 (2016).
13. Letunic, I. & Bork, P. Interactive Tree Of Life (iTOL): an online tool for phylogenetic tree display and annotation. *Bioinformatics* **23**, 127–128 (2007).
14. Wang, M. *et al.* Mass spectrometry searches using MASST. *Nat. Biotechnol.* **38**, 23–26 (2020).
15. Price, M. N., Dehal, P. S. & Arkin, A. P. FastTree 2--approximately maximum-likelihood trees for large alignments. *PLoS One* **5**, e9490 (2010).
