# Supplementary material for: Microbiomes and metabolomes of dominant coral reef primary producers illustrate a potential role for immunolipids in marine symbioses
Source: Commun Biol. 2023 Aug 31;6:896. doi: 10.1038/s42003-023-05230-1 (PMC10471604; doi:10.1038/s42003-023-05230-1)
Supplement: Supplementary file 2 — Description of Additional Supplementary Files [file 42003_2023_5230_MOESM2_ESM.pdf]

Description of Additional Supplementary Files

File name: Supplementary Data 1
Description: P PERMANOVA summary

File name: Supplementary Data 2
Description: Metabolites summary: statistical analysis, Qemistree classification and Mmvec ordination.

File name: Supplementary Data 3
Description: MASST results

File name: Supplementary Data 4
Description: Samples information

File name: Supplementary Data 5
Description: MASST job links in GNPS
