# Supplementary material for: Microbiomes and metabolomes of dominant coral reef primary producers illustrate a potential role for immunolipids in marine symbioses
Source: Commun Biol. 2023 Aug 31;6:896. doi: 10.1038/s42003-023-05230-1 (PMC10471604; doi:10.1038/s42003-023-05230-1)
Supplement: Supplementary file 8 — Reporting Summary [file 42003_2023_5230_MOESM8_ESM.pdf]

nature portfolio

Corresponding author(s): Helena Mannochio-Russo (hmannochiorusso@health.ucsd.edu); Sean O. I. Swift (seanos@hawaii.edu)

Last updated by author(s): Jul 29, 2023

Reporting Summary

Nature Portfolio wishes to improve the reproducibility of the work that we publish. This form provides structure for consistency and transparency in reporting. For further information on Nature Portfolio policies, see our Editorial Policies and the Editorial Policy Checklist.

Statistics

For all statistical analyses, confirm that the following items are present in the figure legend, table legend, main text, or Methods section.

n/a	Confirmed	
☐	☒	The exact sample size (*n*) for each experimental group/condition, given as a discrete number and unit of measurement
☐	☒	A statement on whether measurements were taken from distinct samples or whether the same sample was measured repeatedly
☐	☒	The statistical test(s) used AND whether they are one- or two-sided<br>*Only common tests should be described solely by name; describe more complex techniques in the Methods section.*
☐	☒	A description of all covariates tested
☐	☒	A description of any assumptions or corrections, such as tests of normality and adjustment for multiple comparisons
☐	☒	A full description of the statistical parameters including central tendency (e.g. means) or other basic estimates (e.g. regression coefficient) AND variation (e.g. standard deviation) or associated estimates of uncertainty (e.g. confidence intervals)
☐	☒	For null hypothesis testing, the test statistic (e.g. F, t, r) with confidence intervals, effect sizes, degrees of freedom and P value noted<br>*Give P values as exact values whenever suitable.*
☒	☐	For Bayesian analysis, information on the choice of priors and Markov chain Monte Carlo settings
☒	☐	For hierarchical and complex designs, identification of the appropriate level for tests and full reporting of outcomes
☐	☒	Estimates of effect sizes (e.g. Cohen's *d*, Pearson's *r*), indicating how they were calculated

Our web collection on statistics for biologists contains articles on many of the points above.

Software and code

Policy information about availability of computer code

Data collection	The metabolomics data was acquired on a Vanquish UHPLC system coupled to a Q-Exactive orbitrap mass spectrometer operated by software Thermo Scientific XCalibur. The pooled amplicon library was sequenced using the Illumina Hiseq 2500 platform.
Data analysis	The following list of software and platforms were used for the metabolomics and microbiome analyses analyses: GNPS, MASST, SIRIUS v4.4.1 (including ZODIAC and CSI:FingerID), Classyfire, Qemistree, mmvec v1.0.4, R v4.1.3 and Metaflowmics.

For manuscripts utilizing custom algorithms or software that are central to the research but not yet described in published literature, software must be made available to editors and reviewers. We strongly encourage code deposition in a community repository (e.g. GitHub). See the Nature Portfolio guidelines for submitting code & software for further information.

Data

Policy information about availability of data

All manuscripts must include a data availability statement. This statement should provide the following information, where applicable:

- Accession codes, unique identifiers, or web links for publicly available datasets
- A description of any restrictions on data availability
- For clinical datasets or third party data, please ensure that the statement adheres to our policy

The mass spectrometry data can be accessed on the Mass spectrometry Interactive Virtual Environment (MassIVE) at https://massive.ucsd.edu/ as part of the dataset MSV000085129, which is publicly available. The Feature-Based Molecular Networking and the Qemistree jobs can be accessed online at GNPS under the

following links: https://gnps.ucsd.edu/ProteoSAFe/status.jsp?task=94d7974737ba4a4c82453f11a3ee1a41 and https://gnps.ucsd.edu/ProteoSAFe/status.jsp?task=55a790571af4490fbf7502d44f65e5c7. All the links for the 158 MASST searches performed can be found in the Supplementary Table 1.
Sequence files and sample metadata that support the findings of this study are available from SRA with project number PRJNA701450.
Spatial data used for mapping and processed versions of the metabolite and microbiome data are included in the Github repository https://doi.org/10.5281/zenodo.7388153.

Human research participants

Policy information about studies involving human research participants and Sex and Gender in Research.

Reporting on sex and gender	*Use the terms sex (biological attribute) and gender (shaped by social and cultural circumstances) carefully in order to avoid confusing both terms. Indicate if findings apply to only one sex or gender; describe whether sex and gender were considered in study design whether sex and/or gender was determined based on self-reporting or assigned and methods used. Provide in the source data disaggregated sex and gender data where this information has been collected, and consent has been obtained for sharing of individual-level data; provide overall numbers in this Reporting Summary. Please state if this information has not been collected. Report sex- and gender-based analyses where performed, justify reasons for lack of sex- and gender-based analysis.*
Population characteristics	*Describe the covariate-relevant population characteristics of the human research participants (e.g. age, genotypic information, past and current diagnosis and treatment categories). If you filled out the behavioural & social sciences study design questions and have nothing to add here, write "See above."*
Recruitment	*Describe how participants were recruited. Outline any potential self-selection bias or other biases that may be present and how these are likely to impact results.*
Ethics oversight	*Identify the organization(s) that approved the study protocol.*

Note that full information on the approval of the study protocol must also be provided in the manuscript.

Field-specific reporting

Please select the one below that is the best fit for your research. If you are not sure, read the appropriate sections before making your selection.

☐ Life sciences ☐ Behavioural & social sciences ☒ Ecological, evolutionary & environmental sciences

For a reference copy of the document with all sections, see nature.com/documents/nr-reporting-summary-flat.pdf

Ecological, evolutionary & environmental sciences study design

All studies must disclose on these points even when the disclosure is negative.

Study description	This study is a multi-omic investigation of the metabolites and microbiomes from corals, macroalgae, and crustose coralline algae (CCA) via an intensive, replicated synoptic survey of a single coral reef system (Waimea Bay, O'ahu, Hawai'i). A total of 112 tissue samples were collected.
Research sample	Samples were chosen to include the most dominant types of primary producers in coral reef benthic communities. From the total of 112 tissue samples collected, 41, 45, and 26 samples were relative to CCA, coral, and macroalgae, respectively.
Sampling strategy	The sampling was performed in five benthic sites in a 100m2 region, in which biases were minimized by rapidly sampling triplicate biological specimens of multiple species within each category across a wide area of reef over a 2-day period. With this approach, the sample sizes for each group were sufficient to perform statistical tests for differentiation between different organisms. Samples were collected on dry ice in the field and then lyophilized for long-term storage.
Data collection	Data were acquired following standard protocols for microbiome and metabolomics analyses (as described in the methods section). The LC-MS metabolomics data was acquired by Emily Gentry and Allegra Aron, while the sequencing data was acquired by Genewiz (South Plainfield, NJ, USA).
Timing and spatial scale	Samples were collected between June 20th and 21st (2019) to minimize biases. The sampling was performed in five benthic sites in a 100m2 region. Each site was delineated as a 20 m circle centered on the reported GPS point.
Data exclusions	From the 112 metabolomics data, 9 were judged to be poor quality, clustering closely with method blanks in multivariate space. These were then removed from further analyses.<br>From the sequencing data, microbial ASVs were retained based on the following criteria: Present in >=3 samples at a minimum abundance of 0.001% of the total sample reads and present in >=1 sample at a minimum abundance of >= 0.1% of total sample reads. After filtering, reads in each sample were rarefied down to 15000 reads per sample.
Reproducibility	Samples were randomly allocated in plates for each analysis method to minimize batch effects. Lyophilized sample material, DNA extracts, PCR products, and sequencing libraries were preserved to allow for re-analysis.

Randomization	At each site, scientific divers targeted each of the three primary producer types: Coral, CCA, and macroalgae. For corals and CCA, divers targeted 3 distinct taxa (morphotypes) with 3 replicates of each taxon. For macroalgae, divers targeted up to 6 distinct taxa (morphotypes) with 3 replicates of each taxon. The increased sampling effort for macroalgae was intended to account for the limited number of replicate individuals available within a site for each taxon.
Blinding	Samples were given non-descriptive sample names for data collection and data analysis. Sample groups were only identified at the final data visualization step.

Did the study involve field work? ☒ Yes ☐ No

Field work, collection and transport

Field conditions	Field collections were performed over 2 days in June 2019. The weather was sunny with a daytime temperature of 88 °F. Swell was 1-2 ft.
Location	The location consisted of five collection points named Uppers Beach (21.6389 N, 158.0713 W), Twin Rocks (21.6368 N, 158.0719 W), Sharks Cove (21.6488 N, 158.0657 W), Three Tables Beach (21.6450 N, 158.0666 W), and Waimea Bay (21.6427 N, 158.0663 W).
Access & import/export	Field collection followed the relevant federal and state permitting rules. Collection permit: Hawai'i State Department of Land and Natural Resources Division of Aquatic Resources Special Activity Permit No. 2020-23. Collected samples were processed on island at the University of Hawaii, Mānoa campus. Preserved, non-viable sample material and DNA were exempt from shipping restrictions for both LC-MS analysis and DNA sequencing.
Disturbance	Small portions of material were collected from each individual to allow the organisms to regenerate following sampling. Small chunks of CCA and coral were collected with bone shears while macroalgae were severed above the holdfast to allow for regrowth from the base. The short sampling period of this study minimized impact over time.

Reporting for specific materials, systems and methods

We require information from authors about some types of materials, experimental systems and methods used in many studies. Here, indicate whether each material, system or method listed is relevant to your study. If you are not sure if a list item applies to your research, read the appropriate section before selecting a response.

Materials & experimental systems

n/a	Involved in the study
☒	☐ Antibodies
☒	☐ Eukaryotic cell lines
☒	☐ Palaeontology and archaeology
☒	☐ Animals and other organisms
☒	☐ Clinical data
☒	☐ Dual use research of concern

Methods

n/a	Involved in the study
☒	☐ ChIP-seq
☒	☐ Flow cytometry
☒	☐ MRI-based neuroimaging
